# A Neurocomputational account of the role of contour facilitation in brightness perception

**DOI:** 10.3389/fnhum.2015.00093

**Published:** 2015-02-19

**Authors:** Dražen Domijan

**Affiliations:** Laboratory for Experimental Psychology, Department of Psychology, Faculty of Humanities and Social Sciences, University of RijekaRijeka, Croatia

**Keywords:** assimilation, brightness perception, contrast, computational model, dendrites, lateral inhibition

## Abstract

A new filling-in model is proposed in order to account for challenging brightness illusions, where inducing background elements are spatially separated from the gray target such as dungeon, cube and grating illusions, bullseye display and ring patterns. This model implements the simple idea that neural response to low-contrast contour is enhanced (facilitated) by the presence of collinear or parallel high-contrast contours in its wider neighborhood. Contour facilitation is achieved via dendritic inhibition, which enables the computation of maximum function among inputs to the node. Recurrent application of maximum function leads to the propagation of the neural signal along collinear or parallel contour segments. When a strong global-contour signal is accompanied with a weak local-contour signal at the same location, conditions are met to produce brightness assimilation within the Filling-in Layer. Computer simulations showed that the model correctly predicts brightness appearance in all of the aforementioned illusions as well as in White's effect, Benary's cross, Todorović's illusion, checkerboard contrast, contrast-contrast illusion and various variations of the White's effect. The proposed model offers new insights on how geometric factors (contour colinearity or parallelism), together with contrast magnitude contribute to the brightness perception.

## Introduction

Simultaneous brightness contrast, in which a gray target appears brighter when surrounded by black than a gray target surrounded by white, suggests that brightness perception is not determined solely by the luminance of the target but depends on the luminance of the surrounding region as well. The explanation of brightness contrast involves lateral inhibitory interactions that suppress response near the gray side of the gray-white border and enhance response at gray side of the gray-black border. Differential responses to luminance discontinuities are achieved by center-surround antagonism in the receptive fields found in the early stages of visual processing (Fiorentini et al., 1990). However, lateral inhibition cannot completely account for the perception of brightness. In White's effect (1979; 1981), a gray target that shares a longer border with a white and a shorter border with black, appears brighter than a gray target with the opposite arrangement of borders. Similarly, Benary's cross and Todorović's illusion (1997) show that illusory brightness differentiation also appears when a gray target shares an equally long border with a white and a black background. A further challenge to lateral inhibition is checkerboard contrast (DeValois and DeValois, 1988) and the contrast-contrast effect (Chubb et al., 1989; Cannon and Fullenkamp, 1991).

Table 1 summarizes brightness illusions that are modeled in the present study. All the effects listed in Table 1A involve a gray target which abuts to inducing elements (i.e., stripes, squares). On the other hand, the brightness effects listed in Table 1B including the dungeon illusion (Bressan, 2001), cube illusion (Agostini and Galmonte, 2002), grating illusion (Economou et al., 1998), bullseye display (Bindman and Chubb, 2004a) and ring patterns (Hong and Shevell, 2004; Howe, 2005) illustrate the fact that inducing elements do not need to be adjacent to the target in order to generate brightness illusion. In these examples, a gray target which is completely surrounded by white still appears brighter than a target completely surrounded by black. Therefore, perceived brightness runs in the opposite direction relative to what would be expected from the output of lateral inhibition alone.

**Table 1:** **A list of brightness illusions modeled in the study**.

**A**	**B**
White's effect (1979; 1981)	Dungeon illusion (Bressan, 2001)
Benary's cross (1924)	Cube illusion (Agostini and Galmonte, 2002)
Todorović's illusion (1997)	Grating illusion (Economou et al., 1998)
Checkerboard contrast (DeValois and DeValois, 1988)	Ring patterns (Hong and Shevell, 2004; Howe, 2005)
Contrast-contrast effect (Chubb et al., 1989)	Bullseye display (Bindman and Chubb, 2004a)

A. Brightness illusions where target contours are adjacent to the inducing contours.

B. Brightness illusions where target contours are spatially separated from the inducing contours.

Several computational models were developed in order to account for the brightness effects listed in Table 1 using distinct neural mechanisms. One approach is based on complex receptive field structure (oriented difference of Gaussians—ODOG) accompanied with multi-scale averaging and normalization (Blakeslee and McCourt, 1997, 1999, 2001, 2004). It is possible to derive several versions of the spatial filtering model that are able to simulate a wide range of brightness phenomena (Robinson et al., 2007). However, Economou et al. (2007) noted that the ODOG model cannot account for the dungeon, cube or grating effect as they contain richer geometric structure which is not possible to capture using oriented spatial filters. A further elaboration of the spatial filtering approach is the wavelet decomposition model (Otazu et al., 2008) which is able to explain dungeon illusion but fails with illusions where inducing elements are not of the same size as the target, such as Benary's cross, cube illusion or bullseye display. The reason is that in the model, brightness interactions are possible only within each spatial scale and not across them.

Another approach involves the second-order adaptation mechanism which correctly predicts various types of brightness assimilation effects and their inversions (Barkan et al., 2008). The model first computes second-order opponent responses from which it derives estimates of local and global (or remote) contrast. When the remote contrast is larger than the local contrast, it will adapt local contrast response and produce an assimilation effect. As with the spatial filtering model, this is a multi scale architecture which is not sensitive to structural (geometric) factors. Therefore, it cannot explain the appearance of Benary's cross or Todorović's illusion. Moreover, it cannot predict brightness contrast or the variations of the White's effect constructed by Anderson (2001), Howe (2001) or Yazdanbakhsh et al. (2002). Barkan et al. (2008) suggested that brightness assimilation and contrast have different origins and there is no need to explain them using the same computational mechanisms. It is interesting to note that the predicted magnitude of the assimilation effect is rather small when compared to empirical estimates (Bressan, 2001; Agostini and Galmonte, 2002; Bindman and Chubb, 2004a).

An alternative approach to model brightness perception involves edge integration, where surface brightness is recovered by the spatial summation of logarithms of the local luminance ratios computed at surface edges (Rudd, 2010, 2013). The edge-integration model is inspired by the retinex theory (Land and McCann, 1971), but it incorporates a gain control mechanism which assigns different weights to different edge responses depending on their location, mutual distance and attentional or top-down influences. Rudd (2010) showed that this model can simultaneously account for contrast and assimilation effects in a disk-annulus display. Also, it provides a precise quantitative fit of parabolic relationship observed for lightness matches when the luminance of disk and annulus are systematically varied (Rudd and Zemach, 2004, 2005, 2007). Recently, Rudd (2014) extended the model to include the image segmentation mechanism which enables the modeling of the effect of perceptual grouping on brightness. However, the assumption of this model is that image segmentation occurs only after top-down modulation of the edge response takes place. This is in contrast with empirical evidence that some forms of grouping and figure-ground segmentation can occur without attention (Kimchi and Peterson, 2008; Kimchi, 2009; Shomstein et al., 2010).

Related to edge integration is the filling-in mechanism in which surface brightness is obtained by the lateral spreading of neural signals computed at surface edges (Pessoa et al., 1998; Komatsu, 2006). According to Grossberg and Todorović (1988) perceived brightness arises from the interaction between two parallel processing streams called Boundary Contour System (BCS) and Feature Contour System (FCS). FCS employs lateral inhibition in order to suppress luminance signals across homogenous regions (surface interiors) and enhances signals near luminance discontinuities (surface borders). In doing so, it also computes luminance ratios along the neighboring surfaces, thus solving the problem of brightness constancy. Based on the output from FCS, BCS localizes the surface's boundaries using oriented receptive fields similar to simple and complex cells found in the primary visual cortex. The output of both systems is combined within the filling-in stage where BCS signals serve as barriers to the anisotropic activity spreading of FCS signals. When the filling-in converges to its steady state, its activity is isomorphic to perceived brightness. Properties of FCS and BCS were sufficient to explain how brightness constancy, contrast and assimilation can simultaneously co-occur within the same model, but it was not sufficient to explain the illusions shown in Table 1.

In order to provide a coherent account of brightness illusions that defy explanation in terms of simple lateral inhibition, Ross and Pessoa (2000) extended the Grossberg and Todorović's model to include more elaborate version of BCS which is sensitive to the occurrence of T-junctions. T-junction signals the presence of occlusion where the top of the T is part of the occluding surface while the stem is part of the occluded surface. Ross and Pessoa (2000) suggested that the impact of BCS on the filling-in can be modulated by the grouping signals generated by T-junction detectors. In particular, contrast signals along the stem of the T-junction were attenuated while the contrast signals along the top of the T-junction are preserved, creating the illusory percept. In a similar vein, Kelly and Grossberg (2000) proposed that White's effect and similar illusions arise as a consequence of depth separation where BCS detaches the top of the T-junction from its stem. When surfaces are segregated into different depth layers, target brightness is selectively influenced only by the lateral inhibition arising from the same depth plane. A similar idea has been proposed by Todorović (1997). Both filling-in models were successful in simulating the effects listed in Table 1A, but they cannot handle the illusions listed in Table 1B, suggesting that T-junctions or depth separation cannot be the primary mechanism in generating these brightness effects.

In the present work, a neurocomputational account is developed to explain how contour facilitation can influence the computation of target brightness. An important insight is that in all illusions listed in Table 1, contours of the target are aligned (either collinearly or in parallel) with the contours of inducing elements. Previously, Zemach and Rudd (2007) found that the colinearity of edges present in the surrounding area has a strong impact on the brightness of the target. The only difference between the illusions listed in Tables 1A, B is that in the former, target and inducing contours are connected to each other, while in the latter they are separated by a gap. A further observation is that the contrast of the inducing contour is higher compared to the contrast of the target contour in all the illusions listed in Table 1. A similar idea has been expressed by Anderson (1997) but the scission account also requires the preservation of contrast polarity which is a necessary condition for the perception of transparency to occur. However, the scission model cannot account for the bullseye effect (Bindman and Chubb, 2004a) or ring patterns (Hong and Shevell, 2004; Howe, 2005) where contrast polarity is not preserved among neighboring parallel contours.

The main hypothesis offered here is that the existence of a high-contrast edge which is aligned with a low-contrast edge promotes collinear or parallel contour facilitation within BCS which simulates the properties of orientation-selective units in the visual cortex. Contour facilitation is modeled by the enhanced neural response to low-contrast edge that is aligned with high-contrast edge relative to the response to low-contrast edge that is not. The proposed model suggests that contour facilitation actually attenuates the output of BCS to the filling-in process in FCS. The effect of BCS output attenuation is to allow the filling-in of brightness signals specifically across those edges where contour facilitation occurs in BCS. This activity spreading in FCS simulates the effect of assimilation which opposes the effect of the local contrast computed along the low-contrast edge. Assimilation will not occur across high-contrast edge or across low-contrast edge which is not collinear or parallel with high-contrast edge.

## Model description

The description of the neural architecture for brightness perception is divided into four sections. In the first section, a novel cortical microcircuit is presented which is able to compute recurrent maximum (MAX) function. In the proposed model, the recurrent MAX function achieves two computational goals. In the BCS, it supports contour facilitation among collinear or parallel like-oriented nodes. In the FCS, it supports brightness filling-in among un-oriented nodes. The second section describes the model's components, including two separate processing streams: BCS and FCS. The third section describes the output of the model's components when they reach a steady-state. Finally, the fourth section discusses behavioral and neurophysiological data relevant for contour facilitation.

### Computing MAX function

**Figure 1:**
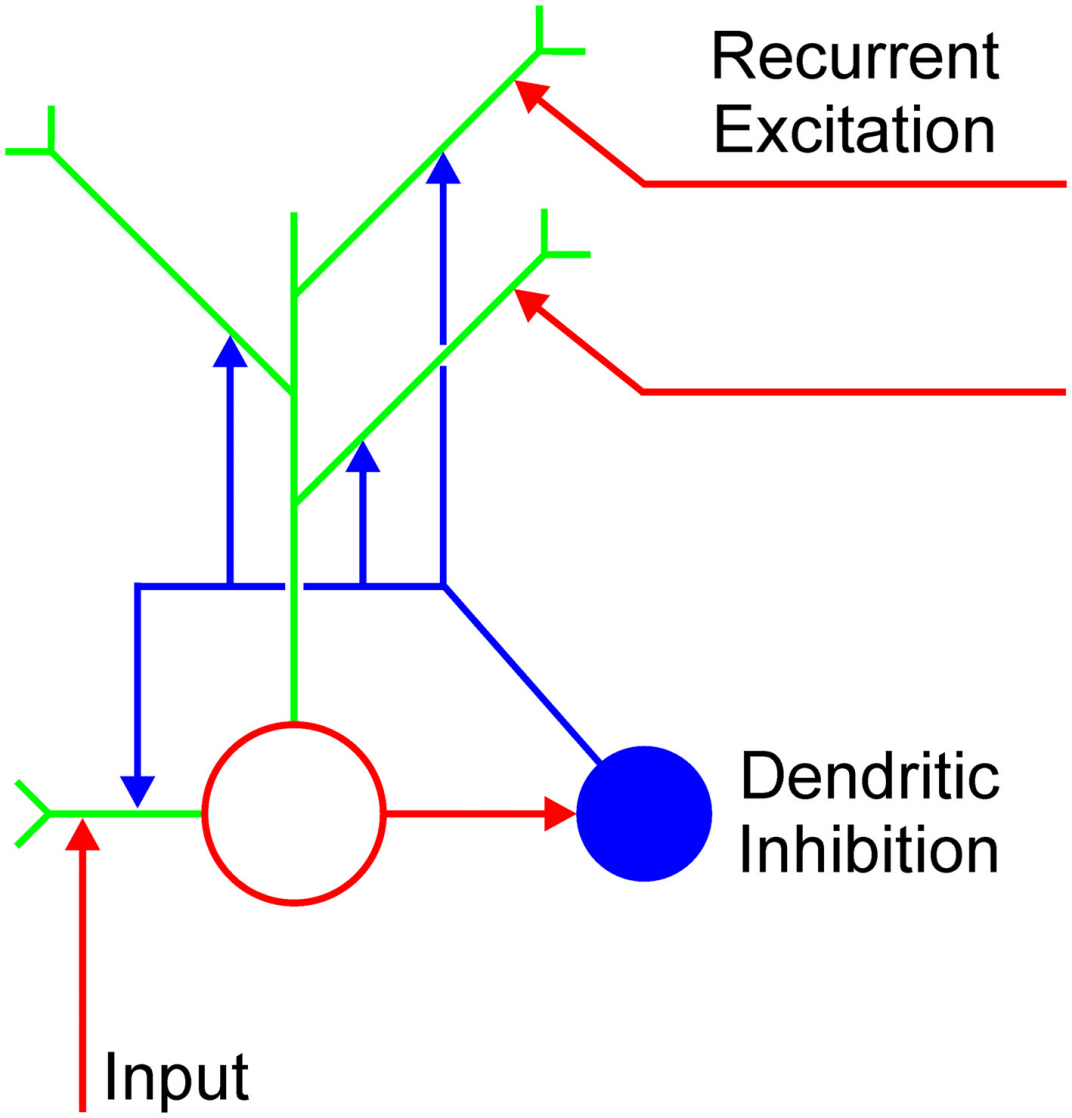
**Cortical microcircuit for computing MAX function**. Open red circle is the excitatory node and blue disk represents the inhibitory interneuron. Green lines are individual dendrites of the excitatory node. Vertical red arrow represents the feedforward excitatory input. Horizontal red arrows pointing to the dendrites represent recurrent excitation arising from the local neighborhood.

The cortical microcircuit capable of computing MAX function is presented in Figure 1 mathematical description of the circuit is given in Appendix A (Supplementary Material). It consists of the excitatory and inhibitory node in a specific synaptic arrangement. The excitatory node is assumed to be a multi-compartment unit with dendrites as independent computational subunits (Häusser and Mel, 2003; Spruston and Kath, 2004; London and Häusser, 2005; Spruston, 2008; Domijan, 2011). The excitatory node receives feedforward input and recurrent excitation from other excitatory nodes encoding the same stimulus property (e.g., contour orientation) at different network locations. Importantly, each recurrent collateral projects to a distinct dendritic branch. Furthermore, the excitatory node sends its output back to the same excitatory nodes from which it receives recurrent collaterals and it also connects to the nearby inhibitory node. Although the excitatory and the inhibitory node are recurrently connected, the inhibitory node does not directly contact the soma of the excitatory node; rather it branches and contacts the dendrites of the excitatory node. In this way, the inhibitory node prevents excessive excitation to arrive at the excitatory node. Recurrent excitation will be able to influence the excitatory node only if it carries a stronger signal compared to the momentary activity level of the target node. The net effect of these interactions is that all mutually connected excitatory nodes will converge to the activity level of the node which receives the strongest feedforward input. Consequently, they will compute the MAX function of their input. In other words, computation of the MAX function emerges as a dynamic property of the neural interactions that control recurrent excitation.

The arrangement of nodes depicted in Figure 1 is supported by anatomical studies showing that axons of a special class of inhibitory cells known as Martinotti cells avoid soma and innervate the dendrites of nearby pyramidal cells (Somogyi et al., 1998; Markram et al., 2004; Kapfer et al., 2007; Silberberg and Markram, 2007). In this way, Martinotti cells create an inhibitory feedback loop which regulates the excitability of the pyramidal cells. Moreover, it was shown that dendritic inhibition mediated by the Martinotti cells is widespread in the cortex and it occurs in the visual cortex as well (Berger et al., 2009). Furthermore, computational analyses and experimental measurements confirmed that the location of an inhibitory synapse on an excitatory cell is an important factor which determines how excitatory and inhibitory inputs are integrated (Liu, 2004; Hao et al., 2009). In particular, the inhibitory synapse on a distal dendrite affects only excitatory input arriving on the same dendrite, but it cannot inhibit excitatory input arriving on other dendrites or excitation arriving on the soma. On the other hand, inhibitory input on the soma has a global effect, that is, it affects the cell's total activity.

### Neural architecture for brightness perception

**Figure 2:**
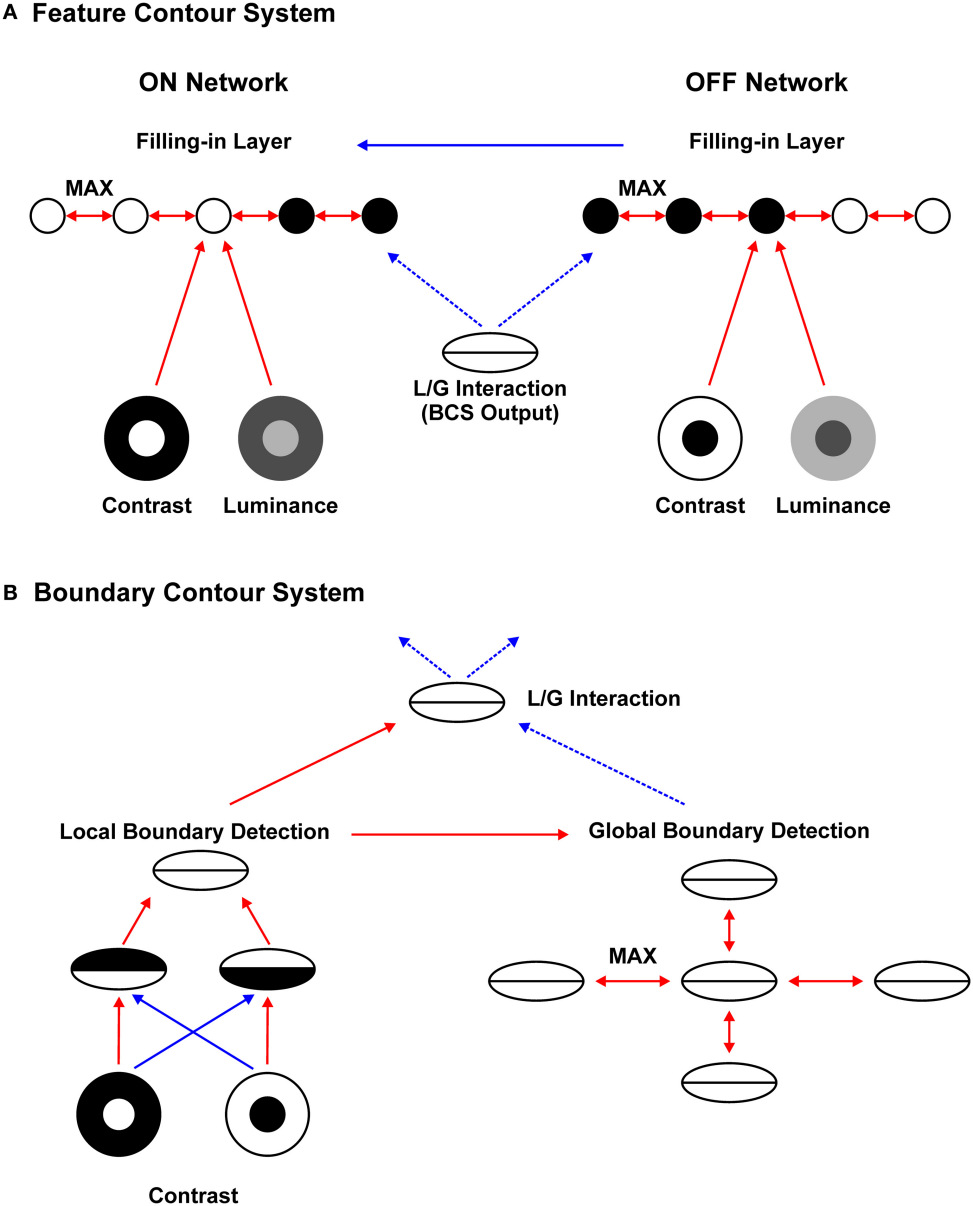
**Neural architecture for brightness perception**. Red arrows represent excitatory connection, full blue arrows denote subtractive inhibition, and dashed blue arrows denote divisive inhibition. Arrows connecting different networks represent one-to-one mapping between them. The Feature Contour System **(A)** consists of the parallel ON and OFF Contrast Pathways, ON and OFF Luminance Pathways and ON and OFF Filling-in Layers. Within the Filling-in Layers, computation of MAX function among nearest neighbors enables activity spreading which recovers surface brightness (ON filling-in) and darkness (OFF filling-in). Activity spreading is prevented by divisive inhibition arising from the Boundary Contour System **(B)**. The BCS consists of the Local Boundary Detection that includes a pair of like-oriented simple nodes with opposite contrast polarities and a complex node that sums their output. The complex node projects to the Global Boundary Detection and to the L/G Interaction stage. Within the Global Boundary Detection, the computation of MAX function between like-oriented collinear or parallel nodes enables signal enhancement for low-contrast Boundary Contour responses. The L/G Interaction computes the ratio between Local and Global Boundary Detection signals. Finally, the L/G Interaction provides divisive inhibition to the FCS.

The proposed neural architecture for brightness perception is depicted in Figure 2A mathematical description of the model is given in Appendix B (Supplementary Material). Consistent with the previous versions of the filling-in approach (Grossberg and Todorović, 1988; Ross and Pessoa, 2000), the current model starts with the division between parallel processing streams: (A) the FCS and; (B) the BCS. Components of FCS and their interactions are shown in Figure 2A. FCS is further divided into two parallel subsystems: ON and OFF Networks. The ON Network simulates the perception of brightness because its output positively correlates with input luminance. On the other hand, the OFF Network simulates the perception of darkness because its output negatively correlates with input luminance (Rudd and Arrington, 2001). This division is reminiscent of parallel on and off channels in the visual system (Schiller, 1992). Within each subsystem, there are three components: Contrast Pathway, Luminance Pathway and Filling-in Layer.

**ON and OFF Contrast Pathways** compute luminance ratios between two sides of luminance border using shunting or divisive inhibition. Computation of the ratio is implemented by the nodes with balanced center-surround receptive fields simulating the properties of neurons in the retina and LGN. These cells respond strongly to sharp luminance borders but their response is attenuated on uniform luminance areas or on shallow luminance gradients. The nodes in the ON Contrast Pathway respond strongly on the bright side of the bright-dark border while the nodes in the OFF Contrast Pathway provide a complementary signal by providing a strong response on the dark side of the bright-dark border.

**ON and OFF Luminance Pathways** encode input luminance and its inverse, respectively. Sensitivity to luminance is implemented by cells with unbalanced center-surround receptive fields in which the center outweighs the surround, thus creating an approximate luminance detector. This model stage is justified by the analysis of Mach bands, Chevreul illusion and the perception of luminance gradients which cannot be accounted for by the output from the nodes with balanced receptive fields (Pessoa et al., 1995; Neumann, 1996; Todorović, 2006). Therefore, the Luminance Pathways provide complementary signals to the Filling-in Layer which are lost within the Contrast Pathways. Such signals are important for implementing the brightness and darkness assimilation described below. The output to the ON (OFF) Filling-in Layer is given by the sum of the neural activity in the ON (OFF) Contrast and Luminance Pathways. The sum is taken separately at each network location.

**ON and OFF Filling-in Layers** integrate the Feature Contour (contrast plus luminance) signals with the output of BCS. The activity of the ON Filling-in Layer is isomorphic to brightness perception while the activity of the OFF Filling-in Layer is isomorphic to darkness perception. Both Filling-in Layers implement the recurrent computation of the MAX function among the nearest neighbor nodes. This slow isotropic spreading of neural activity requires more time to cover larger distances consistent with behavioral (Paradiso and Nakayama, 1991; Paradiso and Hahn, 1996) and neurophysiological data (Hung et al., 2007; Huang and Paradiso, 2008). Activity spreading is blocked by divisive inhibition provided by the output of BCS described below. The output of BCS ensures that the representation of surfaces with different input luminance will remain segregated within the Filling-in Layer. After the Filling-in Layers reach their steady-state, the brightness percept is obtained by subtracting the output of the OFF Filling-in Layer from the output of the ON Filling-in Layer and normalizing it.

Furthermore, nodes in the ON and OFF Filling-in Layers have a threshold that prevents weak luminance signals to start the activity spreading. This threshold ensures that brightness assimilation cannot occur over the black or dark gray surfaces in the ON Filling-in Layer. This constraint prevents the spurious brightening of black surfaces. The same threshold in the OFF Filling-in Layer prevents darkness assimilation to occur over white or light gray surfaces. Consequently, when the appropriate conditions are met for assimilation to occur, the ON Filling-in Layer will produce filling-in (brightness assimilation) from the white background to the gray targets, but it will not allow filling-in from the gray targets to the black background. In the same way, the OFF Filling-in Layer will produce filling-in (darkness assimilation) from the black background to the gray targets but it will not allow filling-in from the gray targets to the white background.

Figure 2B depicts the components of the BCS and their interactions. **Local Boundary Detection (LBD)** receives input from the ON and OFF Contrast Pathways. It simulates the orientation selectivity of simple and complex cells found in the primary visual cortex. The receptive field of the model simple node consists of adjacent excitatory and inhibitory lobes elongated along the preferred orientation of the node. Excitatory lobe receives excitatory projections from the ON Contrast Pathway and inhibitory projections from the OFF Contrast Pathway. On the other hand, inhibitory lobe receives excitatory projections from the OFF Contrast Pathway and inhibitory projections from the ON Contrast Pathway. At each network location, there are two simple nodes with opposite contrast polarities, that is, with opposite arrangements of the excitatory and inhibitory lobes. The receptive field of the model complex node is created by summing output from the pair of like-oriented simple nodes with opposite contrast polarities. Consequently, complex cell loses sensitivity to contrast polarity.

Furthermore, the complex node computes the feedforward MAX function among its nearest neighbors (not shown in Figure 2B). In this way, more uniform Local Boundary representation is achieved without attenuation at corners and junctions. Attenuation of the Local Boundary response at corners, junctions, and line ends is a complex issue which is solved within the FACADE theory using inhibitory interactions between hypercomplex cells of the same and of the orthogonal orientations (Gove et al., 1995; Grossberg, 2003). In the present work, such complex interactions are avoided and replaced by the computation of the MAX function between nearby complex nodes.

**Global Boundary Detection (GBD)** receives excitatory projections from the like-oriented nodes in the LBD stage. The GBD consists of nodes capable of long-range collinear and parallel contour facilitation. As Figure 2B suggests, collinear projections are longer than parallel ones, implying that the extra-classical receptive field of the node is larger in the collinear compared to the parallel direction. The GBD nodes implement the recurrent computation of the MAX function which enables activity spreading among the like-oriented nodes in the collinear and to a smaller degree in the parallel direction.

In contrast to the isotropic activity spreading within the Filling-in Layer, orientation-specific activity spreading among GBD nodes is a fast process because they have large extra-classical receptive fields capable of detecting distal input far from their classical receptive field. Consequently, activity spreading within the GBD stage could be achieved in a few steps. It should be noted that the GBD nodes do not have capabilities of bipole cells in the FACADE theory that can generate perception of illusory contours (Gove et al., 1995; Grossberg, 2003). Therefore, there are no GBD signals occurring on the empty space between the collinear or parallel contours.

**Local/Global (L/G) Interaction** represents the output of BCS which is projected to FCS. The L/G Interaction receives excitatory projections from the LBD nodes and divisive inhibition from the GBD nodes. Therefore, it computes the ratio between the corresponding Local and Global Boundary signals at each network location. When the Local and Global Boundary signals are of equal strength, their ratio will be close to 1. This is the case when there is no collinear or parallel contour facilitation among the GBD nodes. On the other hand, when the GBD signal is much larger relative to the LBD, their ratio will be much smaller than 1. The output of the L/G Interaction stage is binary. It will assume value 1 if the computed ratio crosses the threshold and value 0 if the ratio is smaller than the threshold. The threshold is set in a way to favor larger ratios (i.e., ratios closer to 1). In this way, Boundary Contour representation of the low-contrast edge that is collinear or parallel with high-contrast edge will be removed from the BCS output. On the other hand, Boundary Contour representation of the low-contrast edge that is not collinear or parallel with high-contrast edge will remain intact in the BCS output.

### Model's output

**Figure 3:**
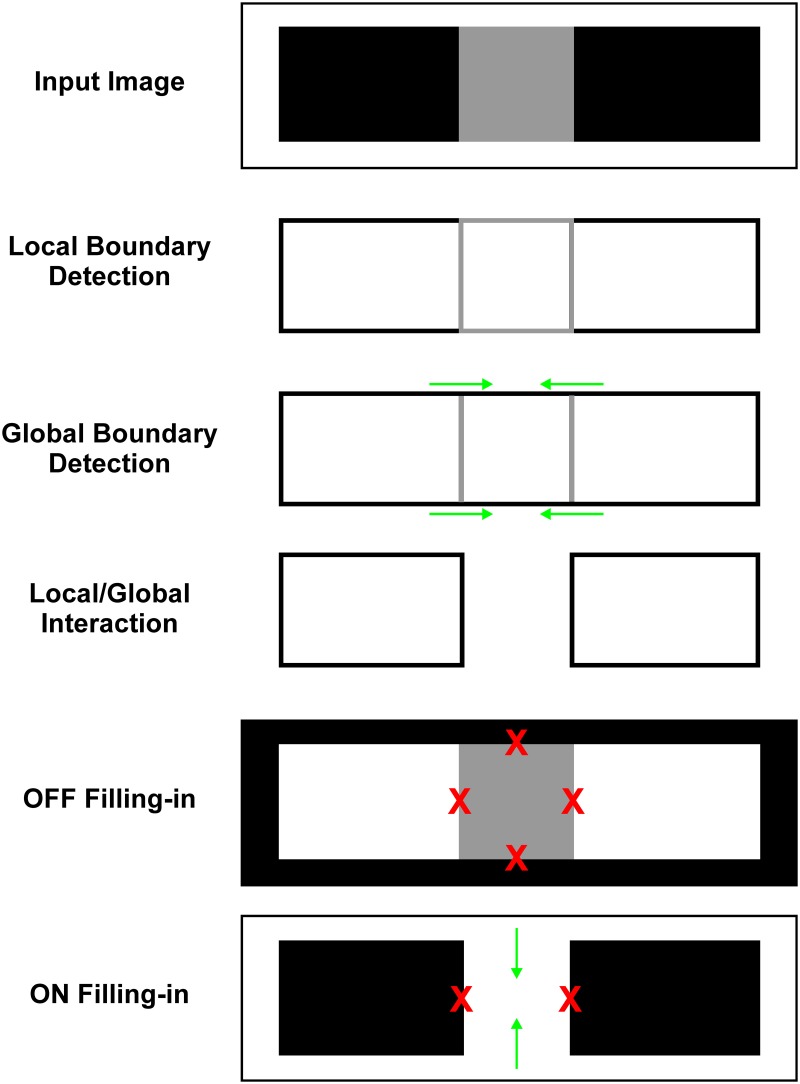
**Schematic illustration of how interaction between the output of the Local and Global Boundary Detection removes the representation of low-contrast edge from the BCS output which triggers assimilation within the Filling-in Layer**. Input image consists of a gray square positioned between two flanking black rectangles circumscribed by the white background. In the LBD, strong signals (black lines) are observed along the black-white edges and weak boundary signals (gray lines) are observed along the black-gray or white-gray edges of the gray square. In the GBD, green arrows depict contour facilitation which enhances Boundary Contour responses along the horizontal edges of the gray square. Ratio between the Local and Global Boundary signals (L/G Interaction) is lower for horizontal edges than for vertical edges of the gray square. Consequently, threshold for L/G Interaction removes the Boundary Contour representation of the horizontal edges of the gray square but leaves representation of its vertical edges intact. In the OFF Filling-in Layer, there is no assimilation along vertical direction because it cannot occur over black region. On the other hand, activity spreading in horizontal direction is prevented by the output of BCS. Blockage of activity spreading is marked by red Xs. In the ON Filling-in Layer assimilation occurs from the white background toward interior of the gray square. Green arrows depict the direction of activity spreading.

In order to better appreciate the image transformations taking place within different network stages, Figure 3 provides a schematic illustration of the output of the major components of the model. Input image consists of gray square flanked by two black rectangles and the whole figure is circumscribed by white background. In BCS, the LBD computes surface borders. Its response is proportional to the contrast magnitude between two sides of the luminance border. Therefore, the black-white edges produce stronger LBD signal relative to the white-gray or black-gray edges of the gray square. The GBD implements contour facilitation (i.e., oriented activity spreading) which enhances weak horizontal Boundary Contour signals at white-gray edge because they are collinear with stronger Boundary Contour signals arising from the white-black edge. On the other hand, there is no contour facilitation for vertical Boundary Contour signals at black-gray border because they are not collinear with white-black border. Consequently, their activity level remains weak in the GBD.

Finally, the L/G Interaction computes the ratio between the output of the Local and Global Boundary Detection stages. This ratio is close to 1 if there is no contour facilitation and it is much smaller at locations where contour facilitation occurs within the GBD. Consequently, L/G interaction will attenuate spatial positions where the Global Boundary signal is much stronger relative to the Local Boundary signal as it occurs on the horizontal edges of the gray square. On the other hand, it will leave intact those locations where the Global and Local Boundary signals are of the same magnitude as it occurs on the white-black edges and on the vertical edges of the gray square. Threshold for the L/G Interaction removes completely Boundary Contour representation of the horizontal edges of gray square. Such removal of the Boundary Contour signals will produce activity spreading (assimilation) within the Filling-in Layers.

In FCS, filling-in enables the brightness signals to spread until they are blocked at the luminance borders by the output of BCS (i.e., the output of L/G Interaction). These Boundary Contour signals are effective in preventing filling-in only when they are complete, that is, when they cover all edges of the surface. In the OFF Filling-in Layer, there is no assimilation because activity spreading is blocked along both horizontal and vertical edges of the gray square. Activity spreading across vertical edges is blocked due to the BCS output. On the other hand, activity spreading across horizontal edges is blocked by the threshold of the Filling-in Layer nodes which prevents activity spreading when they receive weak input. The effect of this threshold is to prevent assimilation across black surfaces. In the ON Filling-in Layer, assimilation occurs from white background to the interior of the gray square due to the lack of the BCS output along the horizontal edges of the gray square. Again, blockage of activity spreading across the vertical edges of the gray square is due to the existing BCS output.

### Empirical evidence for contour facilitation

Model's collinear projections are consistent with psychophysical findings on contour facilitation (Polat and Sagi, 1994) and with anatomical and physiological findings on the long-range horizontal projections in the visual cortex (Spillmann and Werner, 1996; Kapadia et al., 2000; Li and Gilbert, 2002; Roelfsema, 2006). Inclusion of the parallel projections is justified by the psychophysical finding that, in the lateral masking paradigm, the threshold for detection of low-contrast target is reduced when it is surrounded by parallel high-contrast flankers. Although this effect is much smaller when compared to the effect of high-contrast collinear flankers (Polat and Sagi, 1994).

Furthermore, in the path integration paradigm, there is evidence for facilitation among like-oriented Gabor patches positioned in parallel in a so-called “ladder” configuration (Bex et al., 2001; Ledgeway et al., 2005). Anatomical data also suggests that there are excitatory connections among like-oriented cells positioned in parallel beside much more pronounced collinear connections in the primary visual cortex (Bosking et al., 1997). Moreover, the computational analysis of natural image statistics suggests that colinearity and parallelism are both relevant factors (Kruger, 1998). Yen and Finkel (1998) included both, collinear and parallel projections in a model of contour integration in order to achieve better contour detection.

## Results

Computer simulations illustrating the model's behavior are organized into four parts. The first part shows how the model handles illusions where inducing contours are detached from the target but are collinear with the target contours (i.e., dungeon, cube, and grating illusion). The second part shows that the same assimilation effect arises when contours are arranged in parallel (bullseye illusion and ring patterns). Also, it shows that the model's response is reduced to the brightness contrast when there is no contour facilitation between low-contrast and high-contrast contours. Third part shows that the same computational principles explain illusions where inducing elements are connected with the target (i.e., White's effect, Benary's cross, Todorović's illusion, contrast-contrast, and checkerboard contrast). Finally, in the fourth part it is shown that the model is able to explain various modifications of the White's illusions where inducing elements are detached from the target.

### Simulation of long-range contour facilitation

**Figure 4:**
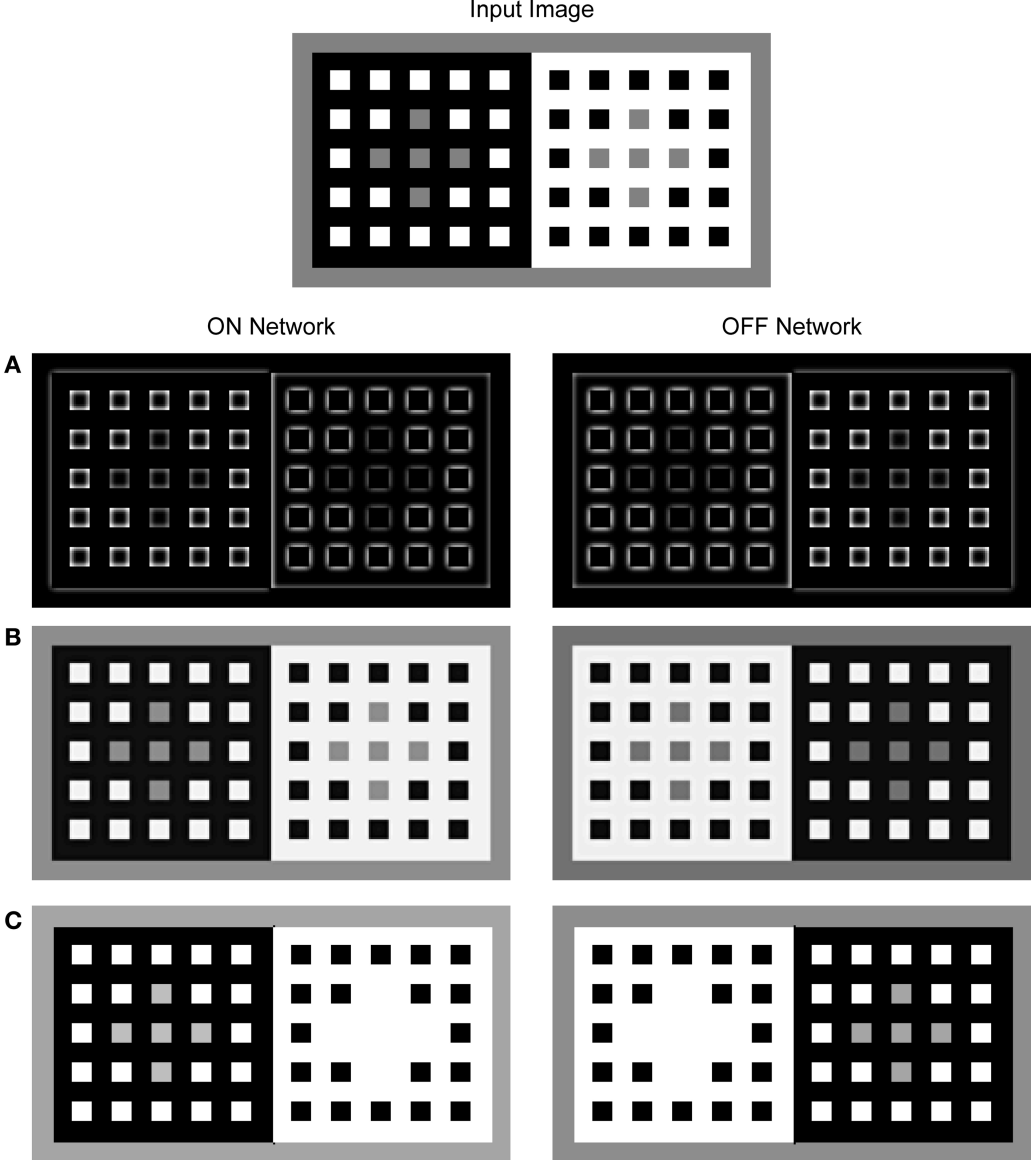
**Neural activity observed for the components of the FCS in a response to the presentation of the dungeon illusion as an input image (Bressan, 2001)**. The activity was mapped onto the brightness code where the minimal activity was labeled by black and maximal activity by white. The output of the Contrast Pathway **(A)**, the Luminance Pathway **(B)**, and the Filling-in Layer **(C)**. The left column shows the response of the ON Network and the right column shows the response of the OFF Network. In the ON Filling-in Layer, assimilation occurs only for the gray squares circumscribed by white. On the other hand, there is no assimilation from the gray squares circumscribed by black because threshold for the activation of filling-in nodes prevents activity spreading across black surface. In the OFF Filling-in Layer, an opposite trend is observed because it encodes an inverse of the input luminance.

The dungeon illusion will serve as an example to illustrate all model stages and to explain how contour facilitation might contribute to brightness perception. Figure 4 depicts components of FCS and their response to the input pattern. The neural activity within the networks was mapped onto the brightness code in a way that the minimal activity was labeled by black and maximal activity by white. The ON and OFF Contrast Pathways showed enhanced response only at luminance borders while their response on the surface's interior is suppressed (Figure 4A). In particular, the ON Contrast Pathway exhibited sensitivity to the brighter side of the luminance border while the OFF Contrast Pathway showed the opposite trend to respond strongly to the darker side of the luminance border. The ON and OFF Luminance Pathways showed enhanced response even over areas of homogenous luminance (Figure 4B). The output of the ON Luminance Pathway is proportional to the input luminance. On the other hand, the output of the OFF Luminance Pathway provides the inverse of the input image; that is, its response is strongest over the areas of low luminance (black surfaces) and weakest over the areas of high luminance (white surfaces).

**Figure 5:**
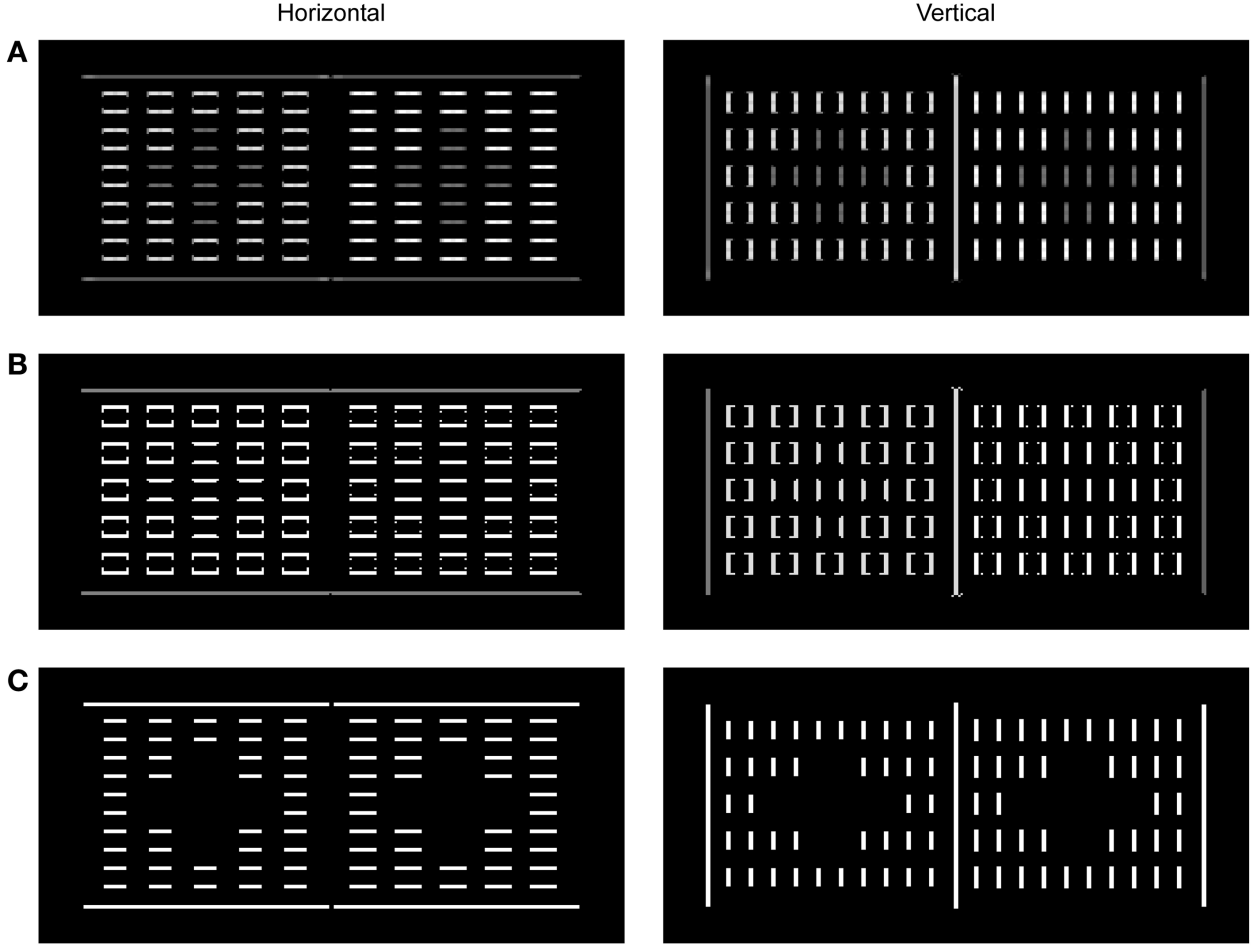
**Neural activity observed for the components of BCS in a response to the presentation of dungeon illusion (Bressan, 2001)**. The activity was mapped onto the brightness code where the minimal activity was labeled by black and maximal activity by white. Output of the Local Boundary Detection **(A)**, the Global Boundary Detection **(B)**, and the L/G Interaction **(C)** are shown for horizontal and vertical orientations, separately. The output of the L/G Interaction shows that the Boundary Contour responses to the gray squares are missing because the ratio between corresponding Local and Global Boundary signals is too small to cross the threshold and to deliver divisive inhibition to the Filling-in Layers. On the other hand, the Boundary Contour responses to small black and white squares as well as to the outer edges of the large black and white surfaces are present because their corresponding Local and Global Boundary Contour signals are of equal strength thus making their ratio sufficiently high to cross the L/G Interaction threshold.

Figure 4C shows the output of the ON and OFF Filling-in Layers. Within the ON Filling-in Layer, the gray squares on the white background completely disappeared from the surface representation. This effect is due to the assimilation of white color that circumscribes the gray squares. Assimilation is a consequence of the fact that there is no appropriate Boundary Contour representation for the gray squares for this input pattern. The output of BCS around gray squares is missing because the LBD signals are much weaker relative to the GBD signals as shown in Figure 5. Therefore, the ratio between the Local and Global Boundary signals is too small to cross the threshold within the L/G Interaction stage.

Interestingly, there is no assimilation for the gray squares on the black background in Figure 4. Here, the activity spreading is prevented by the threshold that controls its start in the Filling-in Layer. Weak luminance signals over the black background cannot cross this threshold and cannot initiate the filling-in. In this way, it is prevented that the simulated appearance of black surface around the gray squares becomes brighter relative to the appearance of black squares surrounded by the white. In the OFF Filling-in Layer, assimilation takes place in the opposite direction. It affects gray squares on the black background, but it does not affect the gray squares on the white background. Here, stronger activity level corresponds to lower input luminance and the reference to black or white in the description is taken with respect to the input image and not with respect to the activity level in the OFF Filling-in Layer. Therefore, the gray squares on the black background (shown as white background within the OFF network) disappeared from surface representation due to the assimilation of black color. On the other hand, the gray squares on the white background remained segregated from their immediate background due to the threshold for initiating the filling-in as mentioned above.

Figure 5 shows the output of the components of the BCS. Based on the output from the ON and OFF Contrast Pathways, the LBD computes the oriented response which simulates the properties of simple and complex nodes (Figure 5A). The output of the LBD is given by the response of the model complex nodes that are not sensitive to contrast polarity. The computation of the feedforward MAX function in the complex nodes achieves uniform Boundary Contour response which is not attenuated at corners or junctions. Importantly, the output of the LBD is sensitive to the contrast magnitude. Therefore, the response is stronger on the black-white edge relative to the gray-black or gray-white edge.

Output from the LBD is projected to the GBD whose output is shown in Figure 5B. These nodes are able to facilitate weak Boundary Contour response if it is collinear or parallel with stronger Boundary Contour response in the neighborhood. As a consequence, initially weak Boundary Contour signals over the gray-black or gray-white edges are elevated to the same activity level as for Boundary Contour signals over the black-white edge. Importantly, the strong Boundary Contour signals over the black-white borders are left intact within the GBD, because recurrent computation of the MAX function is an asymmetrical process. In other words, the nodes with stronger activity could facilitate nodes with weaker activity but the reverse is not possible due to dendritic inhibition. In this way, Boundary Contour signals over the black-white border are the same in the Local and in the GBD.

Finally, the binary output of the L/G Interaction (Figure 5C) shows preserved Boundary Contour signals at locations where the output of the Local and Global Boundary Detection is similar in magnitude. On the other hand, there are no Boundary Contour signals at locations where the output of the GBD is stronger than the output of the LBD. This is the case with the edges of the gray squares. As a consequence, there are no barriers that can prevent activity spreading across the gray squares in the Filling-in Layers as shown in Figure 4C. On the other hand, Boundary Contour representation of the black and white surfaces is complete, that is, it will successfully prevent activity spreading across their edges resulting with unobstructed representation of black and white surfaces in the Filling-in Layers.

**Figure 6:**
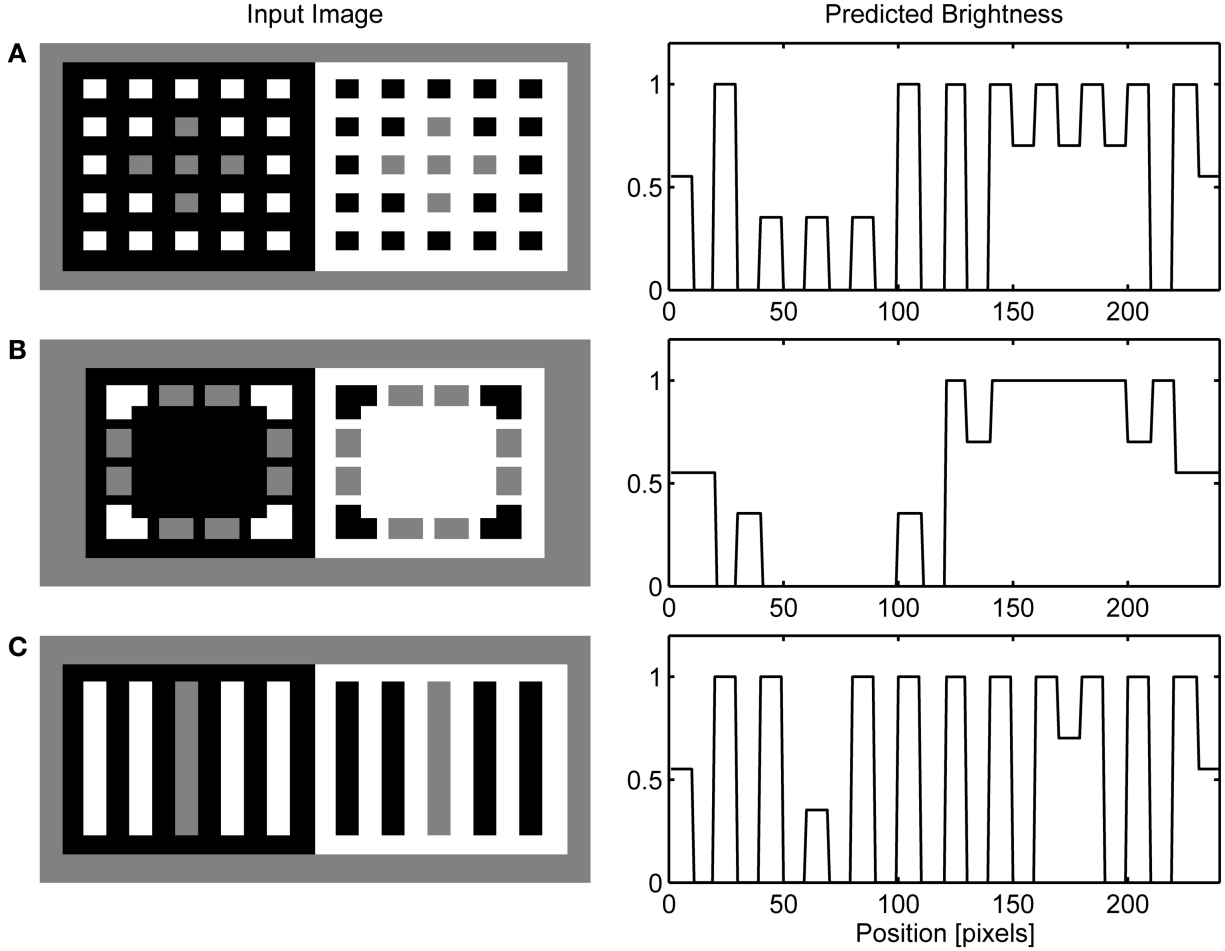
**Simulation of dungeon illusion (A), cube illusion (B) and grating illusion (C)**.

Figure 6A presents the final model's output obtained when the response of the OFF Filling-in Layer is subtracted from the response of the ON Filling-in Layer. I followed the convention of Barkan et al. (2008) to select one row from the input matrix and to display normalized neural activity corresponding to this row. Figure 6 shows that the same computational principles highlighted above for the dungeon illusion can be applied in simulation of the cube (Figure 6B) and grating illusion (Figure 6C). Agostini and Galmonte (2002) argued that their illusion is a consequence of the grouping of gray targets with a black or white Necker cube. This grouping is facilitated by the perception of a 3-D layout of the Necker cube. Moreover, they reported that the effect is stronger when observers experienced the switching between two alternative interpretations of the image. As noted above, the present model does not incorporate mechanisms of depth perception and it cannot account for the impression of depth in the Necker cube nor the reversal between two possible depth interpretations in this figure. However, this model offers a different explanation of the cube illusion which involves colinearity of black and white corners with gray targets. Therefore, I reduced their figure to two squares formed by black and white corners. Even in these reduced conditions, the effect is still present. Moreover, the model makes testable prediction that the illusion will disappear when gray patches are moved relative to the inducing corners in a way that will prevent collinear facilitation.

### Simulation of the parallel facilitation and brightness contrast

**Figure 7:**
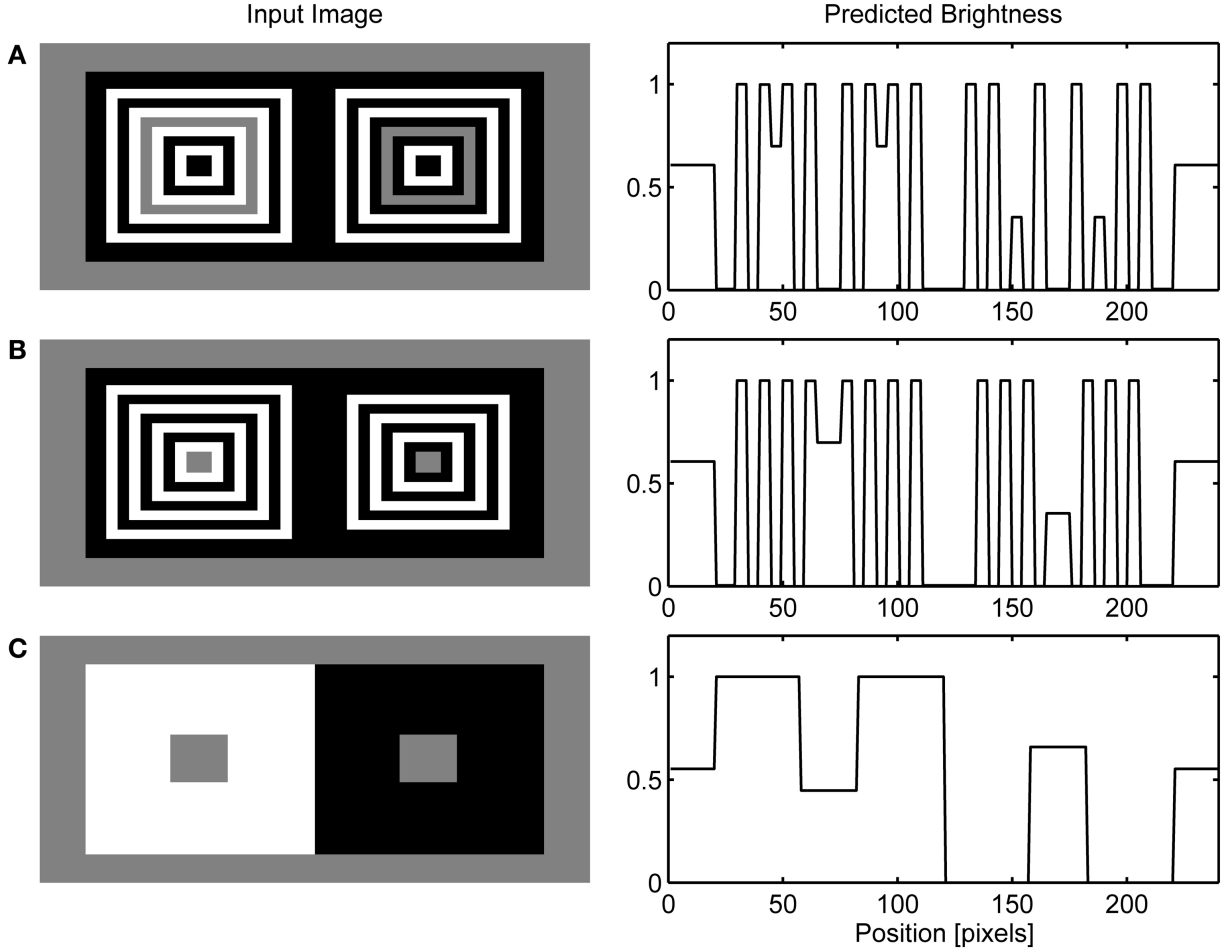
**Simulation of ring patterns (A), bullseye illusion (B) and simultaneous brightness contrast (C)**.

In the previous section, it was shown how collinear contour facilitation can account for the dungeon, cube and grating illusion. However, in the bullseye display (Bindman and Chubb, 2004a) or the ring patterns (Hong and Shevell, 2004; Howe, 2005) there are no high-contrast contours that are collinear with the low-contrast contours of the target gray surface. However, there are parallel high-contrast and low-contrast contours present in these illusions. Figures 7A,B shows that the model can handle these cases also. Due to low resolution, I employed quadratic version of the circular patterns used in the original studies.

The present model accounts for these effects by the observation that when the high-contrast contour is positioned in parallel with the like-oriented low-contrast contour, it will produce the same excitatory effect as collinear contour will do within the GBD. This is a consequence of the fact that the model's GBD nodes have excitatory connections with like-oriented nodes positioned orthogonal to the preferred axis of orientation (Figure 2B). Parallel contour facilitation of low-contrast Boundary Contour response will produce discrepancy in the activity level for LBD and GBD along the edges of gray target. In particular, the GBD signals will become much larger compared to the LBD signals making their ratio too small to cross the threshold in the L/G Interaction stage. The lack of Boundary Contour representation for gray targets induces assimilation from abutting surfaces within the Filling-in Layers. As a result, we will observe brightening of the gray stripe abutting on the white stripes and darkening of the gray stripe abutting on the black stripes (Figure 7A). The same explanation holds for the bullseye illusion (Figure 7B).

Furthermore, Figure 7C shows that when there is no opportunity for collinear or parallel contour facilitation between contour segments of different contrast magnitudes, the model predicts the appearance of brightness contrast because the activity of the Filling-in Layer will be driven by the contrast signals obtained from the center-surround antagonism in the Contrast Pathway. In this case, the Filling-in Layer cannot produce the assimilation effect because there is no difference between the activity levels of LBD and GBD. Therefore, their ratio is close to 1 and the output of the L/G Interaction will prevent filling-in along all edges of the gray targets.

It is interesting to note that the observed magnitude of brightness contrast is weaker compared to Grossberg and Todorović (1988) because the Filling-in Layer combines output from the Contrast and Luminance Pathways where Luminance Pathway lead to more veridical brightness perception. Furthermore, the strength of the brightness contrast relative to the assimilation effects will depend on the weighting of the Contrast relative to the Luminance Pathway signals when they input into the Filling-in Layer. Greater weight on the contrast signal will produce a stronger contrast effect and a weaker assimilation effect. In this model, weights of the Contrast and Luminance Pathway signals were set to a fixed value but it is possible that they vary across individuals which will produce individual difference in the strength of perceived brightness effects. Experimental evidence supports this by the finding that observers who experience a stronger contrast effect report a weaker assimilation effect and vice versa (Robinson et al., 2007).

### Simulations of short-range contour facilitation

**Figure 8:**
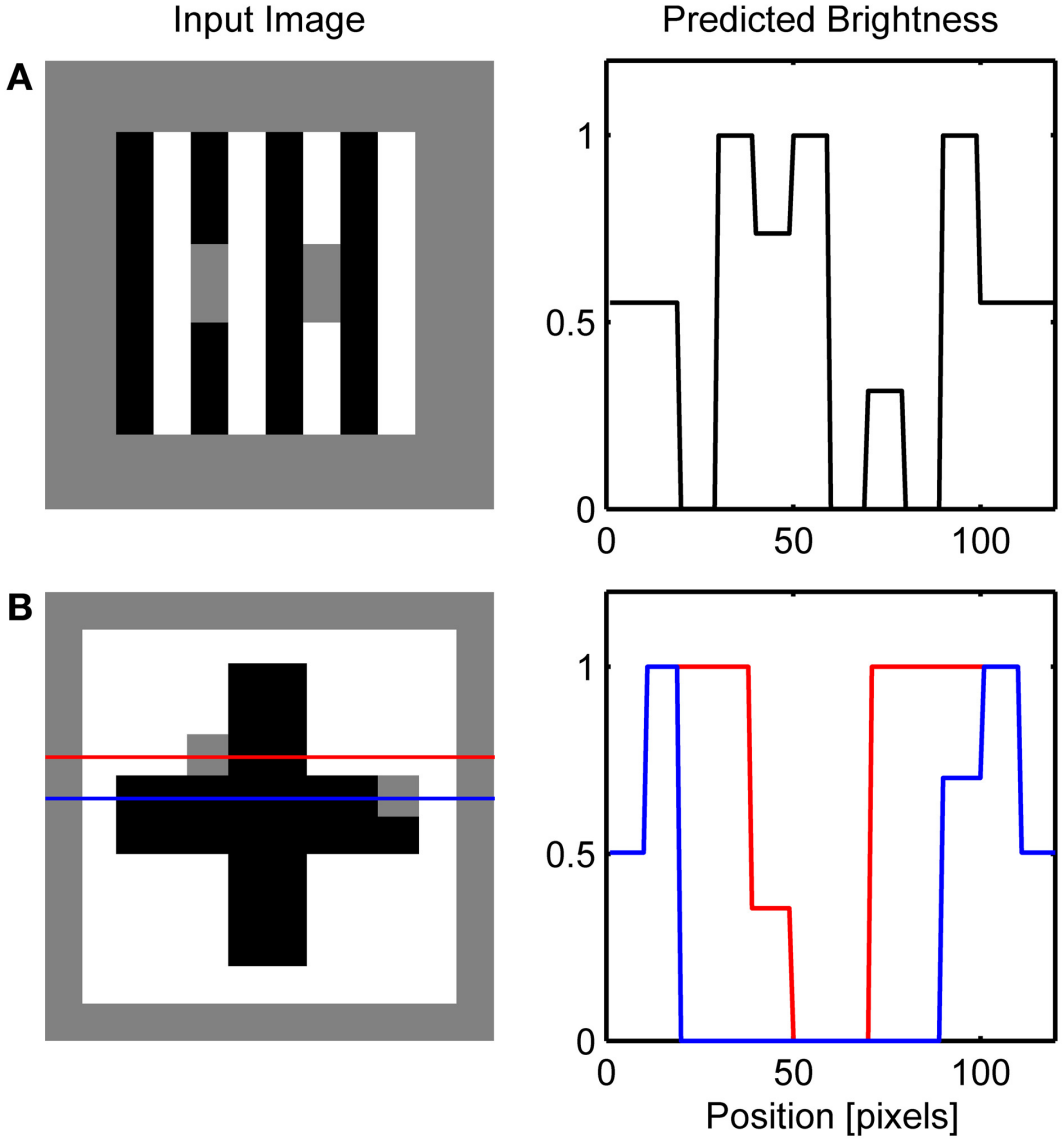
**Simulation of White's effect (A) and Benary's cross (B)**. Red and blue lines on the left depicts row of network nodes whose activity level is chosen for the display on the right.

**Figure 9:**
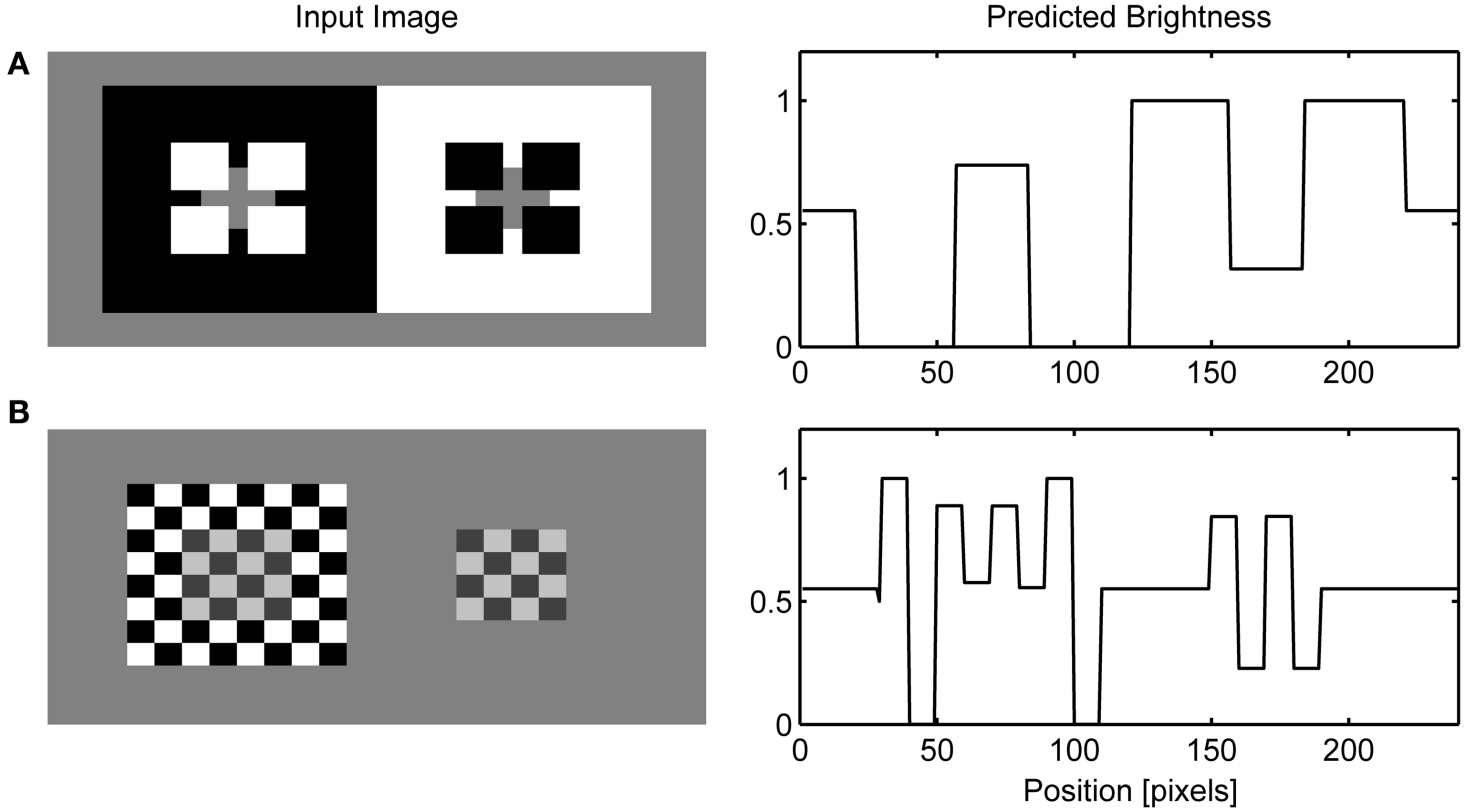
**Simulation of Todorović's illusion (A) and contrast-contrast effect (B)**.

Figure 8 depicts the results of the simulation of White's effect and Benary's cross, while Figure 9 shows the simulation of checkerboard contrast. All these effects share the common feature of the existence of collinear borders of different contrast magnitude. Therefore, the same mechanism of the interaction between the Global and Local Boundary Contour signals that was used to explain dungeon illusion can be extended here to induce assimilation along the low-contrast border and to create illusory brightness difference. In White's effect, brightening of the gray target positioned on the black bar arise from the assimilation of white from the flanking white bars. This effect occurs within the ON Filling-in Layer. On the other hand, the darkening of the gray target on the white bar arises from assimilation from the flanking black bars within the OFF Filling-in Layer. Here, black bars are represented with a higher activity level relative to the gray target which will produce assimilation. In a similar vein, in Benary's cross, the brightening of the gray target on the black cross results from the assimilation from the white background and not from the contrast signals computed as relative to a black cross.

The explanation offered here is closely related to the scission account of the White's illusion proposed by Anderson (1997). However, an important difference is that the present account does not require the preservation of contrast polarity along the collinear borders. The proposed model is insensitive to the contrast polarity due to the fact that the input to the global boundary computation arises from the complex nodes. These nodes are insensitive to the contrast polarity because they add up outputs from the simple nodes sensitive to the same orientation at the same location but with opposite contrast polarities. Limitation of such design choice is that it cannot generate the perception of transparency but the advantage is that it can account for brightness illusions even where there is no accompanied impression of transparency.

**Figure 10:**
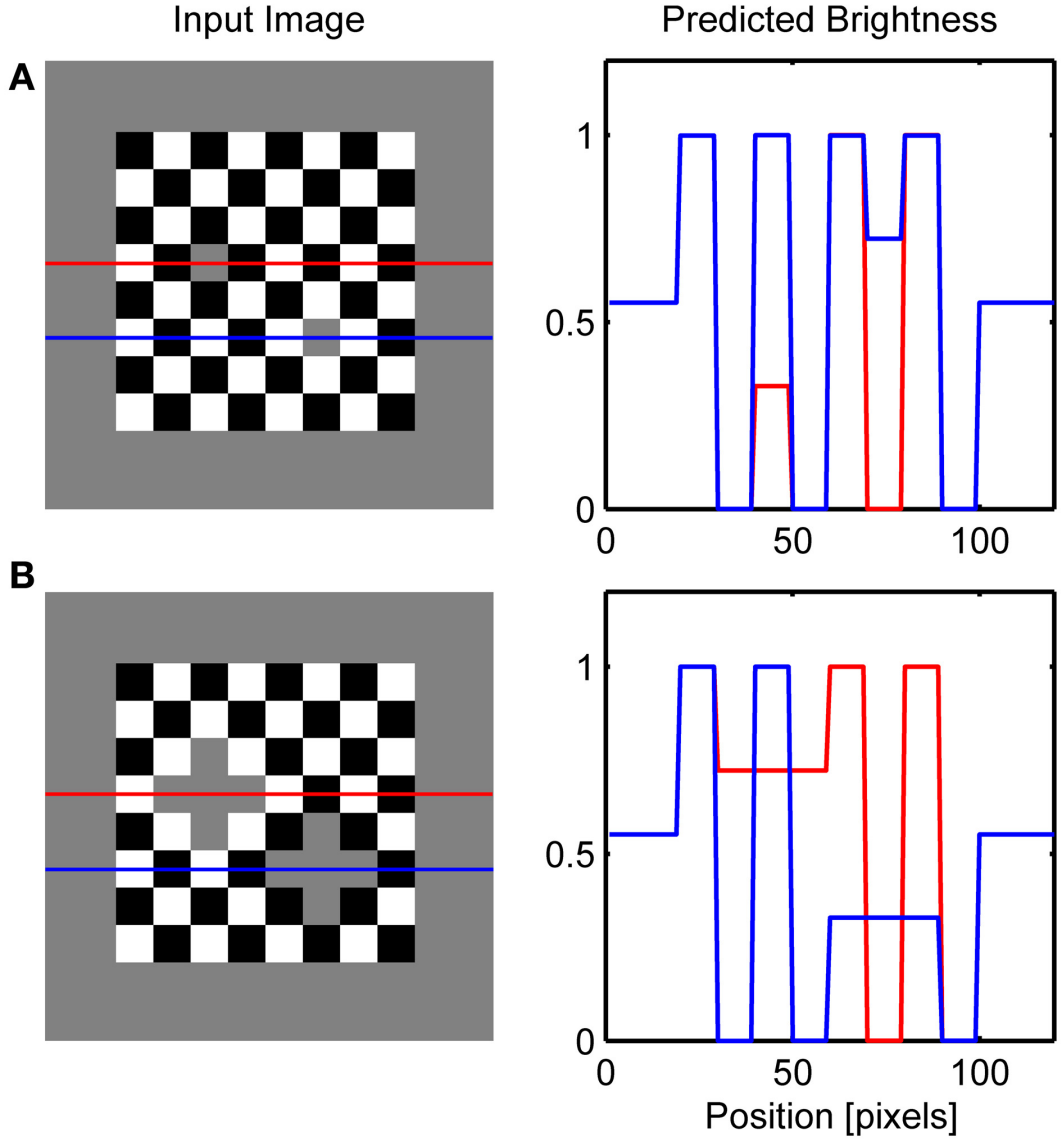
**Simulation of the classic checkerboard contrast (A) and its extended version (B)**. Red and blue lines on the left depicts row of network nodes whose activity level is chosen for the display on the right.

Figure 10A shows that the model correctly simulates Todorović's illusion. It is of particular interest because it motivated the explanation of brightness effects in terms of depth separation (Kelly and Grossberg, 2000; Ross and Pessoa, 2000). For instance, Todorović (1997) suggested that the gray target on the left side is perceptually segregated from the occluding white squares and grouped with the black background. Therefore, contrast signals arising from the gray-white border are given lower weight compared to the gray-black border in the determination of final brightness. The same process occurs on the right side of the image, but here the gray-black border is given lower weight. The computational model that explains how selective integration of contrast signals might occur within the filling-in approach was given by Ross and Pessoa (2000).

Here, an alternative explanation is given because the effect is attributed to the assimilation of surface color from the occluding squares. More precisely, brightening of the gray target on the left is a consequence of the brightness assimilation arising from the white occluding squares and the darkening of the gray target on the right is a consequence of the darkness assimilation arising from the black occluding squares. Güçlü and Farell (2005) provided evidence that assimilation could be the cause of this effect. In the case of contrast-contrast illusion (Figure 10B), assimilation in the central patch of gray squares produces contrast reduction on the left part of the image while there is no such effect on the right because the background is of lower contrast.

### Simulations of the variants of the white's effect

**Figure 11:**
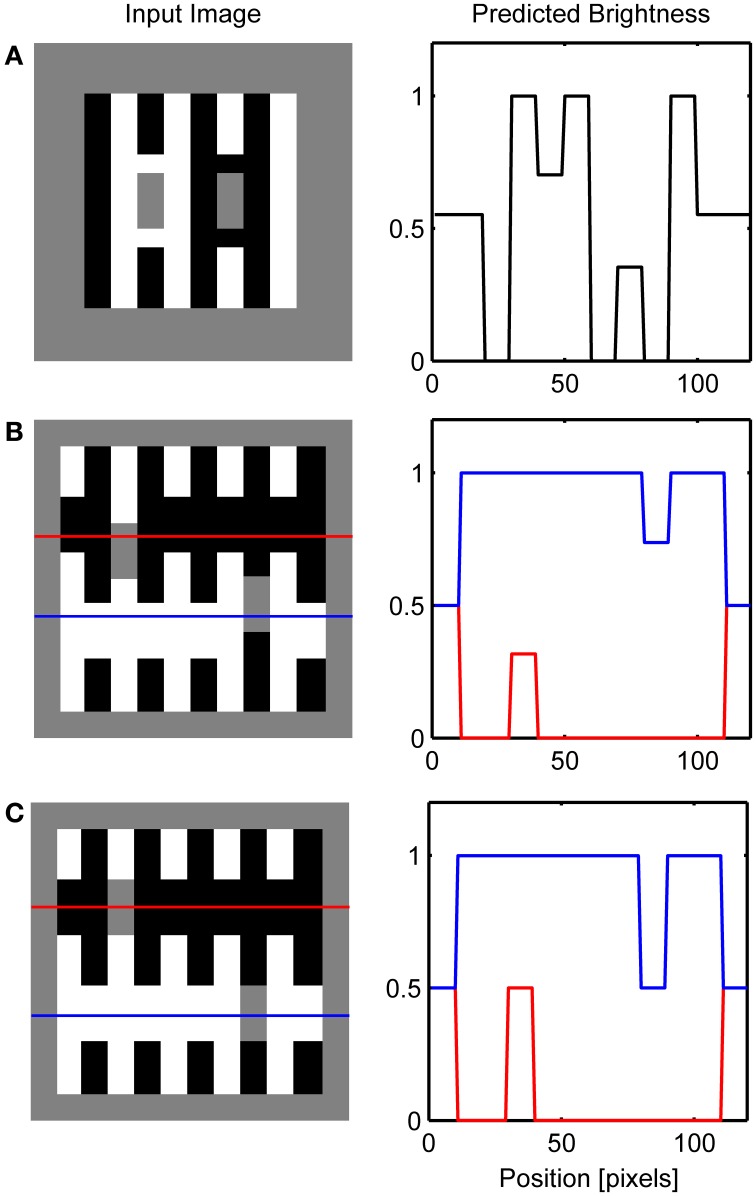
**Simulation of variations on the White's effect including: (A) Yazdanbakhsh et al. (2002), (B) Anderson (2001) and (C) Howe (2001)**. Red and blue lines on the left depicts row of network nodes whose activity level is chosen for the display on the right.

Several variations of White's illusion have been created in order to show that the explanation based on T-junction or transparency is not sufficient to explain the illusion. Simulations of these effects are presented in Figure 11. For instance, Yazdanbakhsh et al. (2002) showed that when the target is detached from the inducing black and white bars the illusion remained with undiminished strength (Figure 11A). Such manipulation is not problematic for the present model because it creates conditions similar to that in dungeon illusion. That is, gray targets are completely surrounded by white or black but distant high-contrast inducing elements create the illusion that the gray target on white is brighter than the gray target on black.

The importance of collinear contour facilitation is illustrated by the variants of White's illusion devised by Anderson (2001) and Howe (2001). When the gray target is translated vertically relative to the inducing grating so that the horizontal grouping is not possible because the borders of the target are too far away (Figure 11B), only vertical grouping is possible resulting in the standard White's effect. Empirical measurements suggest that the magnitude of this illusion is reduced relative to the standard White's effect (Robinson et al., 2007). Such reduction in the size of the brightness effect is not possible to simulate using the current version of the model because the output of BCS is binary, that is, it signals only whether the Boundary Contour signal is present or absent at a particular location.

However, when the gray target is positioned in a way that all of its borders are subject to collinear contour facilitation (Figure 11C), both black-gray and white-gray Boundary Contour signals will be removed from the L/G Interaction output. In this case, vertical contour facilitation is part of the standard White's illusion but there is also a long-range horizontal contour facilitation. Assimilation within the ON Filling-in Layer will produce brightening of the gray target on the left, but assimilation within the OFF Filling-in Layer will produce darkening of the same target. The same effect will appear for a gray target on the right. As a result, the total effect of the assimilation within both ON and OFF Filling-in Layers will cancel each other out and all gray targets will appear approximately equally bright.

## Discussion

A novel filling-in model is developed in order to explain challenging brightness illusions listed in Table 1. The model behavior relies on the operations of dendrites which are modeled as independent computational units. Artificial dendrites enable the model's nodes to compute maximum function. This is achieved through recurrent inhibition where the excitatory node inhibits its own dendrites via a special group of interneurons. Dendritic inhibition enables the node to remain sensitive to the stronger input signals relative to its own activity level but to ignore weaker signals. In this way, neural activity will exhibit a tendency to converge toward the maximal input signal. Recurrent computation of maximum enables the implementation of oriented activity spreading within BCS and isotropic activity spreading within FCS. Oriented activity spreading within BCS achieves contour facilitation which serves as a cue for brightness assimilation. Isotropic activity spreading within the Filling-in Layer of the FCS achieves a slow reconstruction of surface brightness. Therefore, the same computational mechanism can serve different functions within parallel processing streams.

Computer simulations showed that the combined neural activity from the ON and OFF Filling-in Layers correctly predicts the appearance of gray patches in the dungeon, cube, and grating illusion, bullseye display and ring patterns. These illusions are beyond the scope of the previous filling-in models (Kelly and Grossberg, 2000; Ross and Pessoa, 2000) because they do not contain T-junctions as a cue for depth separation. Instead, inducing elements achieve their effect on the target through some unspecified mechanism which either promotes long distance lateral inhibition between the target and inducing elements as suggested by the anchoring theory (Gilchrist et al., 1999; Bressan, 2006) or assimilation with the immediate neighborhood. The present model suggests that the collinear and parallel contour facilitation creates assimilation from the immediate background within the Filling-in Layers. Assimilation will take place at locations where collinear contour facilitation enhances response to the low-contrast contour. At these locations, interaction between the Local and Global Boundary Detection will remove Boundary Contour representation of the low-contrast contour which will result in unconstrained activity spreading in the Filling-in Layer.

Importantly, the same mechanism is also able to account for other brightness illusions such as White's effect, Benary's cross, Todorović's illusion, checkerboard contrast and second-order contrast. Moreover, the model can explain variants of White's effect which do not contain T-junctions such as Yazdanbakhsh et al. (2002). It should be noted that the model also predicts the inverted White's effect. When the luminance of the target is higher compared to the inducing elements, contrast of the contours of the background elements are no longer higher compared to the contrast of the target contours. Consequently, there would be no contour facilitation from the background to the target.

Finally, when the background is homogenous, that is, when there are no inducing elements present in the background, the model generates a simultaneous brightness contrast. This is not the case with the contrast-contrast model which predicts only the assimilation effects Barkan et al. (2008). Although the contrast-contrast model incorporates the mechanism for the effect of remote contrast, this remote influence is “blind” to geometrical factors such as the existence of collinear or parallel contours. On the other hand, the edge-integration model is able to explain contrast and assimilation effects within a common framework using the top-down modulation of edge responses (Rudd, 2010, 2013). Moreover, Rudd (2014) showed how object-based attention can select which edges in complex images will have a greater impact on the computation of target brightness. In the model presented here, the simulation of brightness illusions is achieved without invoking attention or top-down modulations. This model relies on bottom-up factors such as colinearity and parallelism consistent with findings that perceptual grouping and figure-ground segmentation can occur without attention (Kimchi and Peterson, 2008; Kimchi, 2009; Shomstein et al., 2010). Further research is needed to precisely delineate what the role of attention is in generating brightness illusions.

### Limitations

Although the present model is able to account for a wide variety of brightness illusions it has several shortcomings. First, it provides only a qualitative prediction of the direction of the target brightness because the output of BCS is binary. Depending on the stimulus conditions, BCS either allows or prevents assimilation but it cannot modulate its strength. In other words, the model predicts the same magnitude of the assimilation effect for all illusions listed in Table 1 and it is insensitive to factors such as stimulus size or distance between the target and inducing elements (Robinson et al., 2007). As a consequence, the model cannot account for quantitative patterns of lightness matches in disk-annulus display either (Rudd and Zemach, 2004, 2005, 2007; Rudd, 2010). Furthermore, the model is not able to anchor luminance levels to the perceived brightness and to explain the influence of articulation (Economou et al., 2007) or luminance gradients (Zavagno, 1999) on the magnitude of brightness induction. Recently, Grossberg and Hong (2006) proposed a modified version of the Filling-in Layer which is able to anchor luminance values according to the rules of the anchoring theory (Gilchrist et al., 1999).

Finally, the model of the global boundary network includes only excitatory lateral connections although it is known that inhibition plays an equally important role in mediating lateral interactions (Adini et al., 1997). It is possible that effects such as bullseye display or ring pattern arise from lateral inhibition among like-oriented boundary responses as suggested by Bindman and Chubb (2004a). The same mechanism might be necessary to explain assimilation effect observed in Mondrian displays when contrast between the target and background surface is small (Bindman and Chubb, 2004b). However, the addition of inhibitory interactions similar to those proposed by the FACADE theory (Grossberg, 2003) would make the model much more complex. Despite these limitations, the proposed model offers new insights into how contour facilitation can affect brightness perception. In particular, it shows how geometric and photometric factors jointly determine perceived brightness.

### Conflict of interest statement

The author declares that the research was conducted in the absence of any commercial or financial relationships that could be construed as a potential conflict of interest.
