# Supplementary material for: A Neurocomputational account of the role of contour facilitation in brightness perception
Source: Front Hum Neurosci. 2015 Feb 19;9:93. doi: 10.3389/fnhum.2015.00093 (PMC4333805; doi:10.3389/fnhum.2015.00093)
Supplement: Supplementary file 1 [file AppendixA.PDF]

Appendix A. Computing MAX function

Neurophysiological findings suggest that neurons in the visual cortex are capable of computing maximum of their input (Gawne & Martin, 2002; Sato, 1989). Yu et al. (2002) proposed a biophysically plausible neural circuit that computes maximum. Furthermore, Domijan (2003) showed how a single inhibitory interneuron implements the MAX operator using its recurrent projections on the input pathways. However, the goal of these models is to locate the input with maximal activity. They do not have the capacity to propagate the maximal input in a systematic way to other network locations.

Cortical microcircuit depicted in Figure 1 is capable of computing the recurrent MAX function. It consists of mutually connected excitatory and inhibitory node. The excitatory node is modeled as a multi-compartment unit with soma, and basal and apical dendrites as independent computational subunits. The temporal evolution of the electrical activity in the soma of the excitatory node at network location (*i,j*) is given by the non-linear shunting model (Grossberg, 1988)

$$\tau_x \frac{dx_{ij}}{dt} = -\alpha x_{ij} + \left(\beta - x_{ij}\right) D_{ij} . \tag{A.1}$$

In eqn. (A.1), parameter α represents passive decay which drives the activity to the resting state in the absence of the external input, and β is the upper bound of the node's activity. Term D_{ij} represents the total dendritic input to the node described as

$$D_{ij} = \omega_b s_b \left[I_{ij} - x_{ij} \right] + \omega_a h\left[x_{ij} \right] s_a \left\{ \sum_{pq \in M_{ij}} s_d \left[x_{pq} - x_{ij} \right] \right\} . \tag{A.2}$$

In the eqn. (A.2), parameters ω_b and ω_a are dendritic weights that control the strength of the impact of the basal and the apical dendrite on the soma, respectively. The model depicted in Figure 1 distinguishes three independent computational subunits within a single excitatory node: 1) distal dendritic branches of the apical dendrite; 2) trunk of the apical dendrite and the basal dendrite; and 3) the soma (Spruston, 2008). Each subunit performs sigmoid transformation of incoming synaptic inputs and transmits its output to the next subunit in the hierarchy (Häusser & Mel, 2003; Poirazi et al., 2003; Spruston & Kath, 2004). Experimental evidence suggests that individual dendrites can operate as independent decision-making subunits because they can generate dendritic spikes when stimulated by suprathreshold input (Polsky et al., 2004; Wei et al., 2001). Furthermore, dendritic activity is enhanced when the animal is awake suggesting its importance in behavior (Murayama & Larkum, 2009).

Output function *s* of each dendrite (basal or apical) is defined as

$$s\left[x\right] = \begin{cases} S & if \quad x \geq S \\ x & if \quad S > x > 0 \\ 0 & if \quad x \leq 0 \end{cases} \tag{A.3}$$

with *S* as an upper bound. The function s_b describes the output of the basal dendrite where feedforward input arrive from some earlier network stage I_{ij}. The basal dendrite is able to directly activate the soma of the excitatory node. The function s_a describes the output of the trunk of the apical dendrite which integrates the outputs from its distal branches s_d. Branches

of the apical dendrite receive the recurrent excitatory signals arising from the local neighborhood (p,q). An example of the set of locations M_{ij} that will be used in the description of the brightness model is a set of nearest neighbor locations given by

$$M_{ij} = \{(p,q) : 0 < |p-i| + |q-j| \le 2\}. \tag{A.4}$$

Importantly, each recurrent excitatory projection arrives on a distinct branch of the apical dendrite.

Furthermore, all dendrites (basal and apical) receive inhibition from the inhibitory node whose temporal dynamics is not explicitly modeled. Rather, its activity is subsumed under the term $-x_{ij}$ because it is assumed that the inhibitory node reacts quickly to its input and that it faithfully tracks the output of corresponding excitatory node. The inhibitory node simulates the properties of a special class of inhibitory interneurons known as Martinotti cells that receive input from nearby excitatory nodes and specifically target dendrites of these excitatory nodes (Berger et al., 2009; Kapfer et al., 2007; Silberberg & Markram, 2007). Such anatomical arrangement effectively creates inhibitory feedback loop which is capable of computing the function maximum. Function h is a Heaviside step function defined as

$$h[x] = \begin{cases} 1 & if \quad x > 0 \\ 0 & if \quad x \le 0 \end{cases} \tag{A.5}$$

which modulates the influence of the recurrent excitatory signals on the target node. As a consequence, recurrent excitatory signals are modulatory, that is, they can exert their influence on the target node only if the soma of the node is already stimulated by the direct feedforward input. Domijan (2011) provided detailed biophysical arguments for such multiplicative interaction between the soma and the apical dendrite.

In order to avoid time-consuming numerical integration of a set of differential equations, the neural activity of the excitatory node could be approximated by the discrete-time model

$$x_{ij}(t+1) = \max\left\{ x_{ij}(t), h\left[x_{ij}(t)\right] \max_{pq \in M_{ij}} \left[x_{pq}(t)\right] \right\}, \tag{A.6}$$

which is iterated until the steady-state is reached subject to the initial conditions $x_{ij}(0) = I_{ij}$. This approximation is used in the specification of the model of brightness perception given in the Appendix B. Approximation is justified by the observation that according to the eqn. (A.2), activity of the excitatory node will grow, that is

$$\frac{dx_{ij}}{dt} > 0 \quad if \quad x_{ij} < \max_{pq \in M_{ij}} \left[x_{pq}\right] - \alpha, \tag{A.7}$$

and it will reach an equilibrium

$$\frac{dx_{ij}}{dt} = 0 \quad if \quad x_{ij} = \max_{pq \in M_{ij}} \left[x_{pq}\right] - \alpha. \tag{A.8}$$

Computer simulations were run using the eqns. (A.1) and (A.2) in order to verify this approximation with the following set of parameters: $\tau_x = 10$; $\alpha = 0.1$, $\beta = 10$, $\omega_b = 1$, $\omega_a = 0.1$, $S = 10$.

Furthermore, it is possible to compute the MAX function over feedforward input to the excitatory node. In this case, the total dendritic input to the soma is given by

$$D_{ij} = \omega_b s_b \left[I_{ij} - x_{ij} \right] + \omega_a h \left[I_{ij} \right] s_a \left\{ \sum_{pq \in M_{ij}} s_d \left[I_{pq} - x_{ij} \right] \right\}. \tag{A.9}$$

The only difference with respect to the eqn. (A.2) is that the distal branches of the apical dendrite also receive feedforward input from the local neighborhood (p, q) rather than recurrent signals. Discrete approximation of the computation of the feedforward MAX function is given by

$$x_{ij} = \max \left\{ I_{ij}, h \left[I_{ij} \right] \max_{pq \in M_{ij}} \left[I_{pq} \right] \right\}. \tag{A.10}$$

This is a single step computation because there are no recurrent connections present in the eqn. (A.9).
