# Supplementary material for: A Neurocomputational account of the role of contour facilitation in brightness perception
Source: Front Hum Neurosci. 2015 Feb 19;9:93. doi: 10.3389/fnhum.2015.00093 (PMC4333805; doi:10.3389/fnhum.2015.00093)
Supplement: Supplementary file 2 [file AppendixB.PDF]

Appendix B. Neural architecture for brightness perception

Stimulus intensity I_{ij} at position (i,j) may assume values between 1 (black) and 9 (white). The stimulus is first processed by four parallel streams: the ON and OFF Contrast and ON and OFF Luminance Pathways. Each pathway consists of nodes with center-surround antagonism simulating the properties of retina and LGN.

B.1. ON and OFF Contrast Pathways

The contrast pathway detects luminance discontinuities by computing local luminance ratios between neighboring areas. This is achieved using shunting or divisive inhibition coupled with distance dependent interactions. Temporal evolution of the activity of node x_{ij}^{c+} at position (i,j) in the ON Network is given by

$$\frac{dx_{ij}^{(c+)}}{dt} = -\alpha_1 x_{ij}^{(c+)} + \left(\beta_1 - x_{ij}^{(c+)}\right)\sum_{p,q} C_{pqij} I_{pq} - \left(x_{ij}^{(c+)} + \gamma_1\right)\sum_{p,q} S_{pqij} I_{pq} \qquad \text{(A.1)}$$

and for the node x_{ij}^{c-} in the OFF Network

$$\frac{dx_{ij}^{(c-)}}{dt} = -\alpha_1 x_{ij}^{(c-)} + \left(\beta_1 - x_{ij}^{(c-)}\right)\sum_{p,q} S_{pqij} I_{pq} - \left(x_{ij}^{(c-)} + \gamma_1\right)\sum_{p,q} C_{pqij} I_{pq}\,. \qquad \text{(A.2)}$$

Parameter α_1 describes passive decay which drives node's activity towards zero if there is no input; β_1 (γ_1) defines the excitatory (inhibitory) saturation point, that is, an upper (lower) bound for an activity level that can be obtained. The center (surround) part of the receptive field is obtained by convolution of isotropic spatial filter C_{pqij} (S_{pqij}) described as

$$C_{pqij} = C_1 g\left(p,q,i,j,\sigma_c\right), \quad S_{pqij} = S_1 g\left(p,q,i,j,\sigma_s\right) \qquad \text{(A.3)}$$

with the input I_{pq}. Parameter C_1 (S_1) defines the peak response of the center (surround) filter. Peak values of the spatial filters C_1 and S_1 are chosen in a way to create balance between center and surround of the receptive field. In this way, the node's response to the uniform input will be suppressed. Parameter σ_c (σ_s) defines the width of the filter and the two-dimensional Gaussian function g is defined by

$$g\left(p,q,i,j,\sigma\right) = \frac{1}{2\pi\sigma^2}\exp\left\{-\frac{1}{2\sigma^2}\left[\left(p-i\right)^2 + \left(q-j\right)^2\right]\right\}. \qquad \text{(A.4)}$$

At equilibrium, the ON Contrast activity is defined by

$$x_{ij}^{(c+)} = \frac{\sum_{p,q}\left(\beta_1 C_{pqij} - \gamma_1 S_{pqij}\right) I_{pq}}{\alpha_1 + \sum_{p,q}(C_{pqij} + S_{pqij}) I_{pq}}, \qquad \text{(A.5)}$$

and the OFF Vontrast activity is defined by

$$x_{ij}^{(c-)} = \frac{\sum_{p,q}\left(\beta_1 S_{pqij} - \gamma_1 C_{pqij}\right) I_{pq}}{\alpha_1 + \sum_{p,q}(C_{pqij} + S_{pqij}) I_{pq}}. \tag{A.6}$$

Output of the nodes in the ON and OFF Contrast pathways is given by the capital letter X_{ij}

$$X_{ij} = f\left[x_{ij}\right], \tag{A.7}$$

where

$$f[x] = \begin{cases} x & if \quad x > 0 \\ 0 & if \quad x \le 0 \end{cases} \tag{A.8}$$

describes threshold-linear or half-wave rectified output function. The parameters for the ON and OFF Contrast Pathways were set to: α_1 = 100, β_1 = 100, γ_1 = 100, σ_c = 0.5, σ_s = 1.5, C_1 = 1, S_1 = 1.03361.

B.2. ON and OFF Luminance Pathways

The Luminance Pathways encode the stimulus intensity using the similar center-surround antagonism as in the contrast pathway. Therefore, temporal evolution of activity of node in the ON Network x_{ij}^{l+} and in the OFF Network x_{ij}^{l-} are defined analogously as for the Contrast Pathway

$$\frac{dx_{ij}^{(l+)}}{dt} = -\alpha_1 x_{ij}^{(l+)} + \left(\beta_1 - x_{ij}^{(l+)}\right)\sum_{p,q}\overline{C}_{pqij} I_{pq} - \left(x_{ij}^{(l+)} + \gamma_1\right)\sum_{p,q}\overline{S}_{pqij} I_{pq} \tag{A.9}$$

and

$$\frac{dx_{ij}^{(l-)}}{dt} = -\alpha_1 x_{ij}^{(l-)} + \left(\beta_1 - x_{ij}^{(l-)}\right)\sum_{p,q}\overline{S}_{pqij} I_{pq} - \left(x_{ij}^{(l-)} + \gamma_1\right)\sum_{p,q}\overline{C}_{pqij} I_{pq}. \tag{A.10}$$

The only difference relative to the contrast pathways is that the Luminance Pathways employ spatial filter with slightly different shape

$$\overline{C}_{pqij} = C_2 g\left(p,q,i,j,\sigma_c\right), \quad \overline{S}_{pqij} = S_2 g\left(p,q,i,j,\sigma_s\right). \tag{A.11}$$

Peak values of the spatial filters C_2 and S_2 are chosen in a way to create unbalance between center and surround of the receptive field. In particular, C_2 is chosen to be larger than S_2 resulting with stronger center response to the uniform input relative to the surround. Consequently, response of the node will be proportional to the input luminance.

At equilibrium, the ON Luminance activity is defined by

$$x_{ij}^{(l+)} = \frac{\sum_{p,q}\left(\beta_1 \overline{C}_{pqij} - \gamma_1 \overline{S}_{pqij}\right) I_{pq}}{\alpha_1 + \sum_{p,q}(\overline{C}_{pqij} + \overline{S}_{pqij}) I_{pq}} \tag{A.12}$$

and the OFF Luminance activity is defined by

$$x_{ij}^{(l-)} = \frac{\sum_{p,q}\left(\beta_1 \overline{S}_{pqij} - \gamma_1 \overline{C}_{pqij}\right) I_{pq}}{\alpha_1 + \sum_{p,q}(\overline{C}_{pqij} + \overline{S}_{pqij}) I_{pq}}. \tag{A.13}$$

Additionally, the OFF Luminance activity is augmented by the tonic signal J before the output function is applied

$$X_{ij}^{(l-)} = f\left[x_{ij}^{(l-)} + J\right] \tag{A.14}$$

in order to generate above-threshold signal. The parameters for the Luminance Pathways were set to: $\alpha_1 = 100$, $\beta_1 = 100$, $\gamma_1 = 100$, $\sigma_c = 0.5$, $\sigma_s = 1.5$, $C_2 = 3$, $S_2 = 1$, $J = 10$.

B.3. Integration of Contrast and Luminance Pathways

Output from the Contrast and Luminance Pathways are combined or multiplexed separately for the ON Network

$$x_{ij}^{(m+)} = w_1 X_{ij}^{(c+)} + w_2 X_{ij}^{(l+)} \tag{A.15}$$

and for the OFF Network

$$x_{ij}^{(m-)} = w_1 X_{ij}^{(c-)} + w_2 X_{ij}^{(l-)} \tag{A.16}$$

in order to provide Feature Contour signals for the ON and OFF Filling-in Layers, respectively. Weights w_1 and w_2 scale the relative contribution of the contrast and luminance signal in generating brightness percepts, respectively. Higher values of w_1 induce a tendency toward brightness induction while higher values of w_2 induce tendency toward brightness assimilation. In all simulations, weights were set to $w_1 = 3$, and $w_2 = 1$.

B.4. Local Boundary Detection

This model stage simulates the properties of simple and complex cells which are sensitive to boundary orientation. Sensitivity to orientation is achieved by convolution of oriented Gabor filter with the output of the ON and OFF Contrast Pathways. Formal description of simple and complex nodes follows presentations in Gove, Grossberg and Mingolla (1995) and Grossberg and Mingolla (1993).

The simple node is sensitive to contrast polarity thus it has two lobes with opposite polarities defined as

$$A_{ijk} = \sum_{p,q} \left(X_{pq}^{(c+)} - X_{pq}^{(c-)} \right) f \left[G_{ijpq}^{(k)} \right] \tag{A.17}$$

and

$$B_{ijk} = \sum_{p,q} \left(X_{pq}^{(c-)} - X_{pq}^{(c+)} \right) f \left[-G_{ijpq}^{(k)} \right]. \tag{A.18}$$

Each lobe computes the net difference between output of the ON and OFF Contrast Pathways. Function *f* describes half-wave rectification given by eqn. (B.7) and G_{ijpq}^{k} is an odd-symmetric Gabor filter specified by

$$G_{ijpq}^{(k)} = G \sin\left(H W_{ijpq}^{(k)} \right) \exp\left\{ -\frac{1}{2} \left[\left(\frac{U_{ijpq}^{(k)}}{\sigma_1} \right)^2 + \left(\frac{W_{ijpq}^{(k)}}{\sigma_2} \right)^2 \right] \right\} \tag{A.19}$$

where *G* is constant that scales the Gabor amplitude, *H* controls the frequency of the filter's sinusoidal modulation, σ_1 specifies the width of the filter in the preferred orientation, and σ_2 specifies the width of the filter in the orthogonal orientation.

Terms

$$U_{ijpq}^{(k)} = (p-i)\cos\left(\frac{2\pi k}{K} \right) - (q-j)\sin\left(\frac{2\pi k}{K} \right) \tag{A.20}$$

and

$$W_{ijpq}^{(k)} = (p-i)\sin\left(\frac{2\pi k}{K} \right) + (q-j)\cos\left(\frac{2\pi k}{K} \right) \tag{A.21}$$

describe the effect of shifting a receptive field centered at position (0,0) to position (*i*,*j*), rotating it to orientation *k*, and evaluating it at position (*p*,*q*) given that *K* is total number of orientations.

The output of the model simple node x_{ijk}^{1} at location (*i*,*j*) with orientation *k* is given by

$$X_{ijk}^{(1)} = f\left[A_{ijk} + B_{ijk} - \left| A_{ijk} - B_{ijk} \right| - T_1 \right] \tag{A.22}$$

where T_1 is a threshold that removes weak and noisy boundary responses. The activity of the simple nodes with opposite contrast polarity at the same spatial position are summed into a model complex node x_{ijk}^{2} whose response is insensitive to the contrast polarity,

$$x_{ijk}^{(2)} = X_{ijk}^{(1)} + X_{ij(k+K/2)}^{(1)}. \tag{A.23}$$

Parameter *K* defines the total number of orientations of the simple nodes. For simplicity, only horizontal (*k* = 3) and vertical (*k* = 6) orientations were used in the model.

The response of the simple and complex nodes is weakened at corners or junctions, which has long been recognized as an important problem for filling-in models (Gove,

Grossberg & Mingolla, 1995; Grossberg, 2003). One solution is provided by hypercomplex cells whose end-stopping property helps to enhance boundary response at corners and junctions. Another solution is offered here with the introduction of feedforward computation of the MAX function in the complex cells. The feedforward input x_{ijk}^{2} is transformed into enhanced complex node output x_{ijk}^{3} given by

$$x_{ijk}^{(3)} = \max\left\{ x_{ij}^{(2)}, h\left[x_{ijk}^{(2)} \right] \max_{pq \in M_{ij}} \left[x_{pqk}^{(2)} \right] \right\} \tag{A.24}$$

where the form of the MAX computation is taken from the eqn. (A.10). The set of like-oriented nearest neighbors M_{ij} is given by the eqn. (A.4). The parameters for the Local Boundary Detection were set to: $G = 1$, $H = 0.5$, $\sigma_1 = 0.5$, $\sigma_2 = 1.5$, $T_1 = 0.05$, $K = 12$.

B.5. Global Boundary Detection

The goal of this stage is to detect the presence of strong boundary signals that are collinear to or parallel with the weaker boundary signal in the node's receptive field. Nodes for GBD employ the recurrent computation of the MAX function given by eqn. (A.6) in order to achieve sensitivity to high-contrast boundary signal in their extra-classical receptive field. Discrete dynamics of the activity of the global boundary node x_{ijk}^{4} at position (i,j) and with orientation k is defined as

$$x_{ijk}^{(4)}(t+1) = \max\left\{ x_{ij}^{(4)}(t), h\left[x_{ij}^{(3)} \right] \max_{pq \in N_{ijk}} \left[x_{pqk}^{(4)}(t) \right] \right\}. \tag{A.25}$$

In eqn. (B.25) x_{ijk}^{3} is like-oriented feedforward input from the local boundary network at the same position. The set N_{ij} of network locations defined as

$$N_{ijk} = \left\{ (p,q) : \left(0 < |p-i| \le P \right) \vee \left(0 < |q-j| \le Q \right) \right\} \tag{A.26}$$

describes the extra-classical receptive field of the node. Parameters P and Q specifies the elongation of the extra-classical receptive field in the preferred orientation and orthogonal to the preferred orientation respectively. For horizontal orientation, the size of the surround was set to $P = 10$ and $Q = 4$ while for vertical orientation it was set to $P = 4$ and $Q = 10$. In the simulations presented here, it was sufficient to apply 20 time steps in order to reach steady state. Initial conditions were set to

$$x_{ijk}^{(4)}(0) = s\left[x_{ijk}^{(3)} \right] \tag{A.27}$$

where s is a dendritic output function given by eqn. (A.3).

B.6. Local/Global Interaction

Final output of the BCS is obtained by the interaction between the output of the Local and Global Boundary Detection. The interaction takes the form of the ratio computation that can be implemented as divisive inhibition. The output of the L/G Interaction R_{ij} is given by

$$R_{ij} = \sum_k h\left[\frac{x_{ijk}^{(3)}}{F + x_{ijk}^{(4)}} - T_r\right]. \tag{A.28}$$

The sum is taken over all orientations k, h is a binary output function given by the eqn. (A.6), x_{ijk}^3 (x_{ijk}^4) is the output of the Local (Global) Boundary Detection, F controls the precision of the ratio computation, and T_r is a threshold that suppress the L/G Interaction output at locations where the ratio between Local and Global Boundary signals is less than 1. This will be the case at the gray-black or gray-white borders that are collinear or parallel with the black-white border. Parameters for the L/G Interaction were set to: F = 0.01, and T_r = 0.6.

B.7. ON and OFF Filling-in Layers

Filling-in is implemented as a recurrent computation of the MAX function among nearest neighbor locations in the network. Discrete dynamics of the activity of the node x_{ij}^{f+} at position (i,j) in the ON Filling-in Layer is described as

$$x_{ij}^{(f+)}(t+1) = \max\left\{x_{ij}^{(f+)}(t), h\left[x_{ij}^{(m+)} - T_f\right] \max_{pq\in M_{ij}}\left[\frac{x_{pq}^{(f+)}(t)}{1+\varepsilon R_{pq}}\right]\right\}. \tag{A.29}$$

The form of the computation of the MAX function is taken from eqn. (A.6). Threshold T_f assures that filling-in cannot start if the combined luminance and contrast signal x_{ij}^{m+} defined by eqn. (B.15) is too weak at the given location. In this way, assimilation is prevented from the gray target toward black background within the ON Filling-in Layer. This behavior is illustrated in Figure 4C. The recurrent MAX function is computed over the set M_{ij} of nearest neighbors defined by eqn. (A.4). The term R_{pq} describes the output of the L/G Interaction that prevents activity spreading across luminance borders via divisive inhibition. Parameter ε in eqn. (B.29) controls the strength of divisive inhibition. In the same manner, the discrete dynamics of the activity of the node x_{ij}^{f-} in the OFF Filling-in Layer is described by

$$x_{ij}^{(f-)}(t+1) = \max\left\{x_{ij}^{(f-)}(t), h\left[x_{ij}^{(m-)} - T_f\right] \max_{pq\in M_{ij}}\left[\frac{x_{pq}^{(f-)}(t)}{1+\varepsilon R_{pq}}\right]\right\}. \tag{A.30}$$

Iterations in the ON and OFF Filling-in Layers are initialized with

$$x_{ij}^{(f+)}(0) = s\left[x_{ij}^{(m+)}\right] \quad and \quad x_{ij}^{(f-)}(0) = s\left[x_{ij}^{(m-)}\right], \tag{A.31}$$

where s is a dendritic output function given by eqn. (A.3). In all simulations, it was sufficient to run 200 iterations in order to reach steady state which is much faster compared to the filling-in model proposed by Grossberg and Todorović (1988). Parameters for the Filling-in Layers were set to: ε = 10, and T_f = 3.

B.8. Predicted brightness

Final model's prediction of the brightness percept is obtained by subtracting the output of the OFF Filling-in Layer from the output of the ON Filling-in Layer as in

$$x_{ij}^{(b)} = x_{ij}^{(f+)} - x_{ij}^{(f-)} . \tag{A.32}$$

This value is normalized and qualitatively compared to human perception.

B.9. Parameters

Although the proposed neural architecture for brightness perception has many degrees of freedom, there are several parameters critical for the model's success such as the threshold for activation of the Filling-in Layers T_f , threshold for the L/G Interaction T_r, and weights w_1 and w_2 for the Contrast and Luminance Pathways, respectively. However, small variations in the size of these parameters (up to 20% of the chosen value) do not produce qualitative change in the model's behavior. The only exception is the simulation of the checkerboard contrast where smaller values of T_f produce assimilation from light gray squares to dark gray squares making them indistinguishable when they are surrounded by the black and white squares. The parameters for the Local Boundary Detection were chosen in a way to achieve sharp Boundary Contour representation, but even if the LBD response is blurry, the model would achieve contour facilitation in the GBD and reduction of Boundary Contour representation in the L/G Interaction. Finally, it should be emphasized that all simulation results presented in the paper are produced with the same set of parameters.
